# Thyroid hormone-regulated chromatin landscape and transcriptional sensitivity of the pituitary gland

**DOI:** 10.1038/s42003-023-05546-y

**Published:** 2023-12-11

**Authors:** Young-Wook Cho, Yulong Fu, Chen-Che Jeff Huang, Xuefeng Wu, Lily Ng, Kevin A. Kelley, Kristen R. Vella, Anders H. Berg, Anthony N. Hollenberg, Hong Liu, Douglas Forrest

**Affiliations:** 1 Laboratory of Endocrinology and Receptor Biology, National Institute of Diabetes and Digestive and Kidney Diseases, National Institutes of Health, Bethesda, MD 20892 USA; 2 Department of Cell, Developmental and Regenerative Biology, Icahn School of Medicine at Mount Sinai, New York, New York, 10029 USA; 3 Division of Endocrinology, Diabetes and Metabolism, Weill Department of Medicine Weill Cornell Medicine, New York, New York, 10065 USA; 4 Department of Pathology, Cedars Sinai Medical Center, Los Angeles, California, 90048 USA

**Keywords:** Thyroid diseases, Gene regulation

## Abstract

Thyroid hormone (3,5,3’-triiodothyronine, T3) is a key regulator of pituitary gland function. The response to T3 is thought to hinge crucially on interactions of nuclear T3 receptors with enhancers but these sites in pituitary chromatin remain surprisingly obscure. Here, we investigate genome-wide receptor binding in mice using tagged endogenous thyroid hormone receptor β (TRβ) and analyze T3-regulated open chromatin using an anterior pituitary-specific Cre driver (*Thrb*^b2Cre^). Strikingly, T3 regulates histone modifications and chromatin opening primarily at sites that maintain TRβ binding regardless of T3 levels rather than at sites where T3 abolishes or induces de novo binding. These sites associate more frequently with T3-activated than T3-suppressed genes. TRβ-deficiency blunts T3-regulated gene expression, indicating that TRβ confers transcriptional sensitivity. We propose a model of gene activation in which poised receptor-enhancer complexes facilitate adjustable responses to T3 fluctuations, suggesting a genomic basis for T3-dependent pituitary function or pituitary dysfunction in thyroid disorders.

## Abstract (web summary)

A genome-wide study suggests that the response of the pituitary gland to thyroid hormone is regulated by receptor-enhancer complexes that adjust dynamically to hypothyroid and hyperthyroid conditions.

## Introduction

The pituitary gland, a source of hormones that stimulate diverse tissues^1,2^, is highly sensitive to T3. Complex dysfunction of the anterior pituitary can arise in thyroid disorders. For example, hypothyroidism can decrease growth hormone (GH), elevate thyrotropin (thyroid-stimulating hormone, TSH)^3–5^ and variably disturb prolactin (PRL) or gonadotropins (LH, luteinizing hormone, FSH, follicle-stimulating hormone) in humans^3,6^ and mice^7,8^. The sensitivity of the pituitary gland to thyroid status also provides a basis for hormonal tests used for clinical diagnosis or for monitoring of treatments with thyroid hormone^3,9^. However, despite a decades-long quest^10,11^, little is known of the underlying T3 receptor-chromatin interactions and how the receptor adjusts control at these putative enhancers in varying hypothyroid and hyperthyroid conditions.

T3 receptors are nuclear receptor transcription factors encoded by *Thrb* and *Thra* genes. Loss of both genes causes severe pituitary dysfunction with major depletion of GH and elevation of TSH in mice^12,13^. Loss of *Thrb* alone causes more subtle impairment^14–17^ whereas *Thra* mutations cause minimal pituitary phenotypes^18^, suggesting primary control by *Thrb*, although *Thra* partly compensates for many functions. Both *Thrb* and *Thra* are expressed in the anterior pituitary^19,20^. In human resistance to thyroid hormone, *THRB* mutations are associated with pituitary dysfunction^21,22^, although evidence is limited to selected serum hormone measurements with no insights into the genomic responses of the pituitary to T3.

Current views of T3-regulated chromatin in the pituitary gland are largely conjectural, borrowing from in vitro findings, including an intriguing observation that T3 receptors can bind specific DNA elements in the absence or presence of ligand^23,24^. Although a generalized view has emerged in which T3 acts on receptor-enhancer complexes to influence histone modifications and transcription activation^25–27^, the genomic responses to T3 in vivo in the pituitary and most other tissues are undefined. Recent studies of the liver suggest that T3 modifies histones^28,29^ but also stimulates receptor binding to chromatin^30^, potentially to a major extent^31^ suggesting an alternative mode of action by recruitment of receptors to chromatin. In addition, the opening of nucleosome arrays in condensed chromatin during enhancer activation by T3 is undefined in pituitary tissue. In other systems, existing open sites allow access for factors such as glucocorticoid receptor in tumor cells^32^ but chromatin opening itself can be stimulated by so-called pioneer factors^33,34^.

The lack of insight into how T3 controls chromatin in the pituitary gland is due partly to obstacles arising from the small amounts of tissue available and inadequate reagents. Therefore, we have used genetic tagging approaches to investigate chromatin binding and T3-regulated chromatin accessibility in mice. We report that receptor binding in the pituitary is determined at both tissue- and gene-specific levels and that T3 preferentially controls chromatin responses at subsets of sites that display persistent receptor binding rather than T3-dependent receptor binding. These findings suggest a model of pituitary gene activation involving poised receptor-enhancer complexes that respond adjustably to T3.

## Results

### TRβ binding sites in pituitary chromatin

**Fig. 1:**
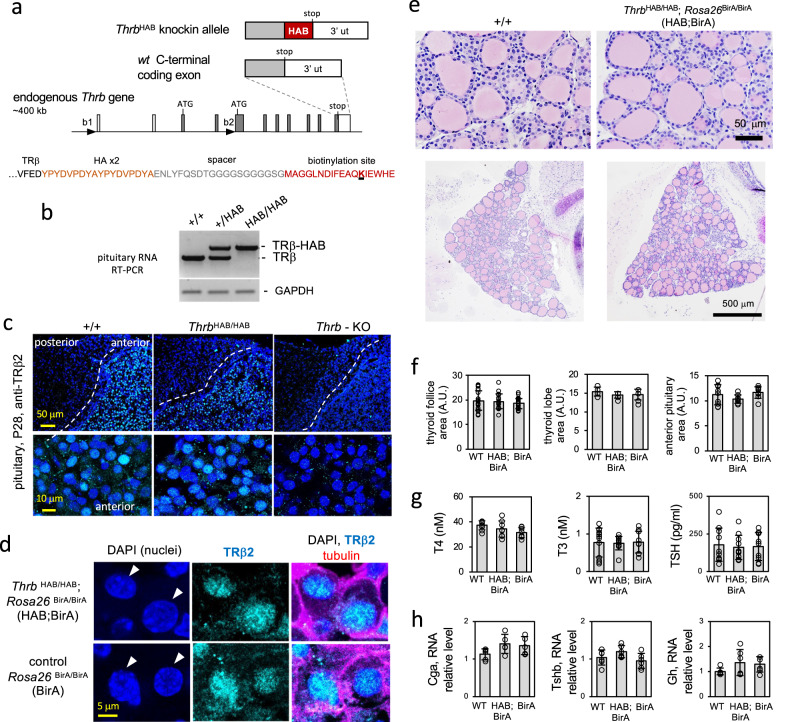
Expression of *Thrb*^HAB^ knockin allele in the pituitary gland. **a**, The HAB tag on TRβ includes an HA epitope and site (target lysine underlined) for biotinylation by BirA ligase (expressed by a *Rosa26*^BirA^ allele). **b** TRβ-HAB transcripts in pituitary RNA detected by PCR. **c** Expression of tagged receptors in pituitary sections detected by immunostaining for TRβ2. *Thrb*-KO, TRβ-deficient control. DAPI, nuclear stain shows tissue background. **d** Immunofluorescence analysis of anterior pituitary sections for tagged (HAB;BirA) and non-tagged receptors (control, BirA) showing nuclear signal detected with TRβ2 immunostaining. Arrowheads, representative nuclei. Alpha-tubulin cytoskeletal stain indicates cell outlines. **e**, **f** Normal thyroid gland morphology and anterior pituitary dimensions in HAB;BirA mice. Thyroid areas were measured on plastic sections. Groups, 4–6 adult males; mean ± SD (*P* > 0.2, one-way ANOVA). Anterior pituitary dimensions were measured for half-lobe areas in cryosections. Groups 4–6 males; mean ± SD (*P* > 0.16, one-way ANOVA). A.U., arbitrary units. **g** Serum total T4, T3 and TSH in adult males; mean ± SD. Groups, 8–12; No significant differences were detected between genotypes (*P* > 0.1, one-way ANOVA). **h** Pituitary gene RNA expression analysis by qPCR. Groups, 5–6 adult males; mean ± SD. No significant differences between genotypes (*P* > 0.1, one-way ANOVA).

To investigate the regulation of the pituitary by T3, we exploited a knockin tag on TRβ to locate receptor-bound chromatin sites in mice. The *Thrb*^HAB^ allele expresses receptors with a tag for biotinylation by BirA ligase that is expressed independently by a *Rosa26*^BirA^ allele^35^(Fig. 1a, b). The tagged receptors are expressed by the endogenous *Thrb* gene in normal tissue patterns and retain a predominantly nuclear localization within the cell, as indicated by immunostaining for TRβ-HAB protein in pituitary sections (Fig. 1c, d). A similar nuclear location for tagged and non-tagged receptors was also supported by western blot analysis of sub-cellular fractions of transfected cells (Supplementary Fig. 1). The tagged receptors mediate normal transactivation with similar sensitivity as non-tagged receptors to T3 in luciferase reporter assays using different types of response element in vitro (Supplementary Fig. 1). We further examined the possibility of phenotypes arising in *Thrb*^HAB/HAB^ mice since *Thrb* mutations can disturb the pituitary-thyroid axis resulting in elevated TSH and thyroid hormones (T3, the active form and thyroxine, T4, a precursor form)^14^. A particularly sensitive indicator of *Thrb* mutations (including knockout, or C-terminal changes that disrupt transactivation by TRβ) is an enlarged thyroid gland (goiter) with distended, colloid-filled follicles^12,14,36^. However, *Thrb*^HAB/HAB^ mice have normal thyroid gland size and follicular histology (Fig. 1e, f) and serum levels of T4, T3 and TSH in normal ranges (Fig. 1g). Anterior pituitary dimensions (Fig. 1f) and RNA levels for TSH subunit (*Tshb*, *Cga*) and growth hormone (*Gh*) genes were in normal ranges (Fig. 1h). Another prominent phenotype for *Thrb* mutations is deafness^37^ but *Thrb*^HAB/HAB^ mice displayed normal auditory thresholds (Supplementary Fig. 1). Although we cannot exclude subtle, undefined alterations in vivo, the lack of obvious phenotypes supports use of *Thrb*^HAB^ mice as a model for screening genomic binding by TRβ. The approach tagged both TRβ1 and TRβ2 proteins encoded by *Thrb* allowing analysis of total receptor function since both isoforms contribute to pituitary function. Individual deletions of TRβ1^17^ and TRβ2^16,38^ are reported to give mild and intermediate hormonal changes, respectively, without goiter, whereas total receptor deletion (*Thrb*-KO) gives pronounced hormonal changes with goiter^12,14,17,39^.

**Fig. 2:**
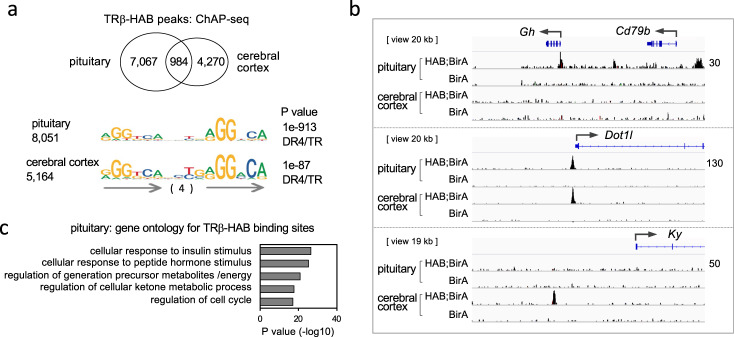
Binding sites for TRβ in pituitary chromatin. **a** TRβ-HAB peaks detected by chromatin-affinity purification (ChAP)-sequencing were identified by differential analysis of HAB;BirA (*Thrb*^HAB/HAB^;*Rosa26*^BirA/BirA^) versus control BirA (*Rosa26*^BirA/BirA^) male mice using SICER (FDR 1e−6). The Venn diagram compares peaks in pituitary and cerebral cortex. Samples contained pituitaries from ~12 mice and cortex from 2 mice; triplicate experiments. The top motifs identified in binding sites by HOMER analysis are direct repeats (arrows) with 4 base spacer (DR4). **b** Examples of TRβ-HAB peaks in pituitary and cerebral cortex. BirA control has very low background in any tissue. Raw reads scale on *right* (same for all tracks for each gene). **c** Gene ontology categories associated with TRβ-HAB binding peaks in pituitary chromatin.

Using high affinity purification, we isolated biotinylated TRβ-HAB protein and associated chromatin from pituitary tissue of *Thrb*^HAB/HAB^;*Rosa26*^BirA/BirA^ (HAB;BirA) mice. Chromatin affinity purification-sequencing (ChAP-seq) identified specific binding sites in HAB;BirA mice compared to control *Rosa26*^BirA/BirA^ (BirA) mice (Fig. 2a, b). BirA control mice had a very low background of non-specific peaks in any tissue. Pituitary binding sites were enriched in the vicinity of genes with ontology categories such as hormonal responses and metabolic functions (Fig. 2c). To indicate the tissue-specificity of TRβ binding in the pituitary, we compared the binding pattern with that in cerbral cortex. Approximately 12% of pituitary peaks were shared with cerebral cortex. For example, a peak was detected in the *Gh* gene (growth hormone) in the pituitary but not cerebral cortex (Fig. 2b). Conversely, a peak was present near the *Ky* gene (kyphoscoliosis peptidase) in cerebral cortex but not pituitary. A shared peak was detected near the *Dot1L* gene (dot1-like histone lysine methyltransferase) in both tissues. The results suggest that major tissue-specific constraints determine receptor binding patterns.

The top consensus motif identified in binding sites in pituitary and cerebral cortex was a direct repeat of AGGTCA with 4 base spacer (DR4), resembling T3 receptor binding motifs first identified in vitro in the *Gh* and other genes^24,40,41^ (Fig. 2a). Although this finding indicates a role for DR4 motifs in receptor binding, the limited overlap of binding sites between tissues suggests that DR4 motifs alone do not determine the tissue-specificity of binding sites. Varied tissue binding patterns are in accord with the regulation of tissue-specific gene networks by T3.

### Regulation of TRβ binding and histone modifications by T3

**Fig. 3:**
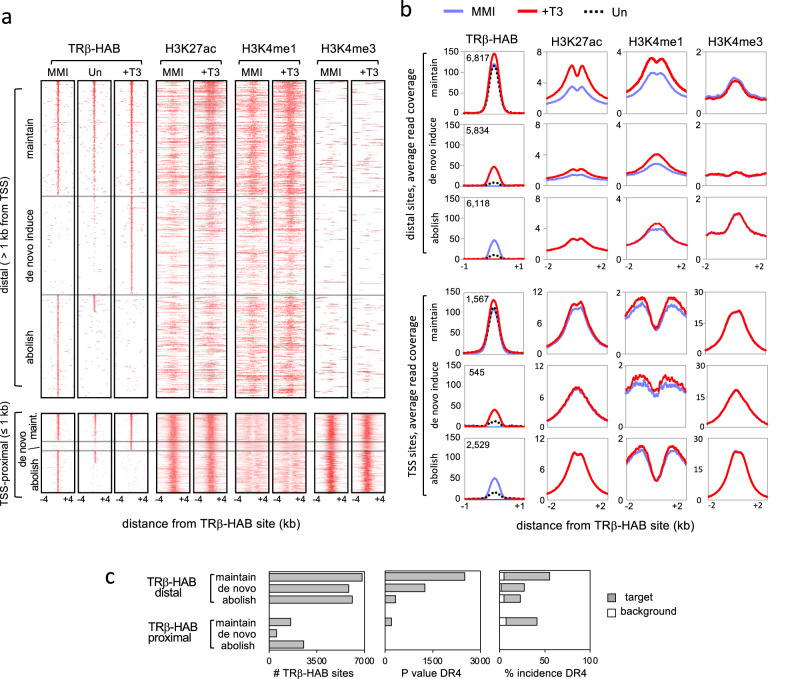
TRβ binding site categories in response to T3 in pituitary chromatin. **a** Genomic heatmaps of TRβ-HAB binding sites in hypothyroid (MMI), hyperthyroid (+T3) and untreated (Un) mice. Peaks were identified by differential analysis of HAB;BirA and BirA datasets (FDR < 1e−6; 50 bp window). Maintained sites were defined as overlapping peaks found in both MMI and +T3 conditions; abolished and de novo sites represented the remaining peaks in MMI and +T3 conditions. In total, 18,769 distal and 4641 TSS-proximal peaks were identified for all three groups. TRβ-HAB peaks are listed from highest to lowest intensity (top to bottom). Peaks for histone marks follow the TRβ-HAB site order. TSS, transcriptional start site. T3-regulated histone marks were detected primarily at distal rather than proximal sites. **b** Average peak profiles reveal T3-induced histone marks notably at distal rather than TSS-located TRβ-HAB sites, particularly in the maintained site category. Total TRβ-HAB peak number is noted in each plot. **c** DR4 was the top statistically significant motif in distal and in proximal maintained binding site categories, identified using HOMER. Additional motifs with lower statistical significance are shown in Supplementary Fig. 3.

In vitro, T3 receptors can bind DNA oligonucleotides in the absence or presence of ligand but in vivo studies have suggested that in liver, receptors are widely recruited de novo to chromatin by stimulation with ligand^31^. To determine whether T3 regulates genomic binding by TRβ in the pituitary, HAB;BirA and control BirA male mouse groups were made hypothyroid by 4–5 weeks of treatment with methimazole (MMI) or hyperthyroid by co-administration of T3 during the final week of treatment (+T3). The treatments established comparatively severe hypothyroid and hyperthyroid conditions in both groups with the goal of maximizing T3-dependent differences for optimal detection of genome-wide responses. Both groups displayed similar, characteristic responses of the pituitary and thyroid glands to MMI and T3 treatments (Supplementary Fig. 2). ChAP-seq revealed 3 approximately equal categories of TRβ binding sites with respect to T3: induced de novo by T3, abolished by T3, or persistently bound at some level regardless of T3 status (i.e., maintained) (Fig. 3a).

To indicate which sites respond functionally to T3, we investigated histone acetylation (H3K27ac) and methylation (H3K4me1) marks as indicators of active enhancers^33^, using chromatin immunoprecipitation-sequencing (ChIP-seq) in hypothyroid and hyperthyroid wild-type mice (C57BL/6J male). T3-increased H3K27ac and H3K4me1 peaks at TRβ binding sites at locations distal (>1 kb) but not proximal (<1 kb) to the transcription start site of nearest genes (Fig. 3a). Notably, T3 regulated these histone marks mainly at sites that maintain TRβ binding rather than sites where T3 abolishes or induces de novo binding (Fig. 3b shows average profile summation plots). This maintained site category shows ~34% increases in TRβ-HAB average read counts in response to T3, suggesting that T3 can moderately augment levels of receptor binding within this category of site. The results also show that T3 primarily increases rather than depletes histone marks at TRβ-bound distal sites, as reduction of H3K27ac or H3K4me1 peaks by T3 was not obvious at the genome-wide scale (Fig. 3a, b). Promoter-proximal sites displayed little or no increase or depletion of H3K27ac or H3Kme1 marks. H3K4me3, a promoter mark, showed little response to T3 at any site.

Under these treatments, DR4 was identified as the top, statistically significant motif at TRβ binding sites with T3-inducible histone marks (i.e., primarily distal, maintained sites) (Fig. 3c). DR4 motifs had low statistical significance at site categories that lacked T3-responsive histone modifications.

TRβ-HAB binding patterns analyzed under narrower ranges of T3 exposure showed that the maintained category of sites constitutes the predominant group of occupied sites in untreated conditions (Fig. 3a). In contrast, few TRβ-bound peaks in the abolished or de novo induced categories were detected in untreated conditions, suggesting that the maintained group, typically at distal enhancer locations, represents the most relevant sites in physiological thyroid hormone ranges.

### Dynamic regulation of chromatin opening by T3

**Fig. 4:**
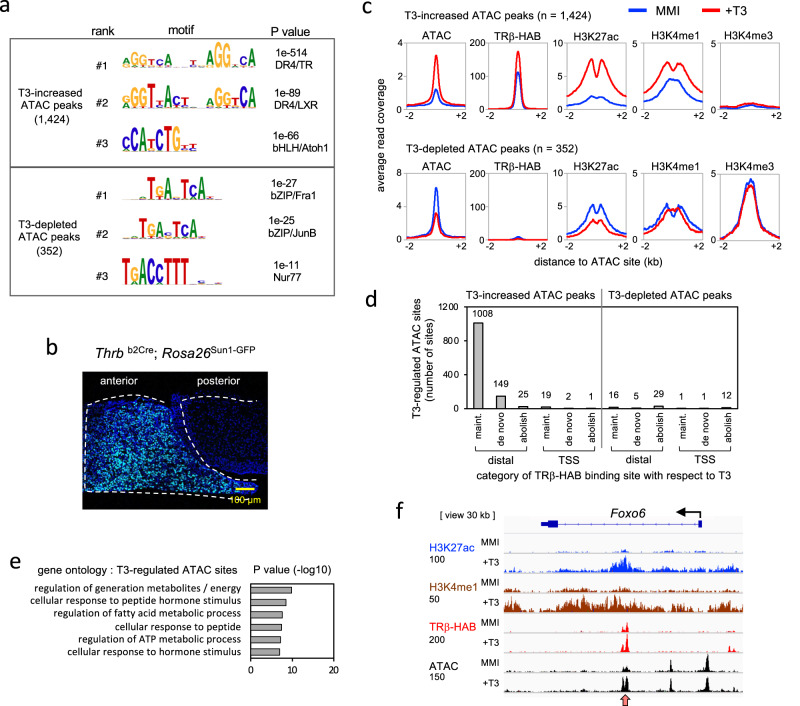
T3-regulated open chromatin in the pituitary gland. **a** ATAC-seq analysis of T3-regulated open chromatin sites in nuclei immunopurified from anterior pituitary of hypothyroid (MMI) and hyperthyroid (+T3) adult male mice. Motifs identified using HOMER analysis. Peaks identified by differential analysis of MMI versus +T3 datasets; cutoff for T3-dependence assigned as ≥ 1.5-fold change in magnitude with adjusted *P* value = 0.05 (SICER analysis). Pooled samples from 3 mice; 4–5 experiments. **b** Nuclei were immunopurified using a nuclear fluorescent protein induced from *Rosa26*^Sun1-GFP^ using a *Thrb*^b2Cre^ driver in the anterior pituitary. **c** Average profile curves of TRβ-HAB binding and histone modifications associated with T3-regulated ATAC peaks. **d** Association of T3-regulated ATAC peaks with TRβ-HAB binding site categories. Number of ATAC peaks noted above columns. **e** Gene ontology associated with T3-regulated ATAC sites. **f** An example gene, *Foxo6*, displays a T3-increased ATAC peak, T3-inducible histone marks and TRβ-bound site (red arrowhead). Raw reads scale, on *left*, below each mark label.

As an independent indicator of T3-dependent enhancers, we investigated T3-regulated open chromatin in anterior pituitary tissue using assay by transposase-accessible chromatin-sequencing (ATAC-seq) (Fig. 4a, b). To attain sufficient sensitivity for the detection of T3-induced changes, we immunopurified nuclei using a *Thrb*^b2Cre^ driver that induces a nuclear envelope epitope from the *Rosa26*^Sun1-GFP^ allele^42^. *Thrb*^b2Cre^ expresses Cre from the b2 promoter of the endogenous *Thrb* gene in anterior pituitary cell populations that are immunopositive for hormones including TSH subunits and GH (Supplementary Fig. 3). We identified 1424 T3-increased ATAC peaks and 352 T3-depleted ATAC peaks, suggesting that T3 regulates both the opening and closing of chromatin at putative inducible (positive) and repressible (negative) enhancers, respectively. The top motif with the highest significance identified at T3-increased ATAC peaks was DR4 which was strikingly similar to TRβ binding sites (Fig. 4a). In contrast, at T3-depleted ATAC peaks, DR4 motif were not obvious, suggesting possible indirect control of these sites without involvement of typical receptor binding motifs. The top motifs identified at T3-depleted ATAC sites were for basic-zipper transcription factors such as Fra1 but with low statistical significance values.

We then tested the predicted association of T3-regulated ATAC peaks with TRβ binding and T3-modified histone marks (Fig. 4c). In accord with the motif predictions (Fig. 4a), T3-increased ATAC peaks were highly associated whereas T3-depleted ATAC peaks were minimally associated with TRβ binding. Most T3-increased ATAC peaks (~85%) were associated with TRβ binding sites, predominantly in the maintained category (Fig. 4c, d). These sites displayed major T3-induced increases in H3K27ac and H3K4me1 histone marks (~98- and 23-fold, respectively), suggesting that these sites represented active, T3-inducible positive enhancers. Chromatin opening predicts a clearance of histones at open sites, which was supported by the appearance of a notch at the center of the T3-induced peaks for the histone marks (Fig. 4c). In contrast, most T3-depleted ATAC peaks (~80%) lacked detectable TRβ binding and displayed comparatively little regulation of H3K27ac and H3K4me1 marks. The lack of motifs for T3 receptors suggests that although T3 reduces chromatin accessibility at these putative negative enhancers, this regulation may be indirect. The relatively small numbers of T3-depleted ATAC peaks identified suggest that negative enhancers occur less frequently than positive enhancers.

Gene ontology analysis indicated that T3-regulated ATAC sites were enriched in gene categories such as response to hormones and metabolism (Fig. 4e, Supplementary Tables 1 and 2). Figure 4f illustrates a representative gene, *Foxo6*, with a maintained TRβ binding site, T3-induced chromatin opening and T3-induced histone marks (H3K27ac and H3K4me1). Other gene examples are shown in Supplementary Fig. 4.

### Transcriptional sensitivity of the pituitary gland determined by TRβ

**Fig. 5:**
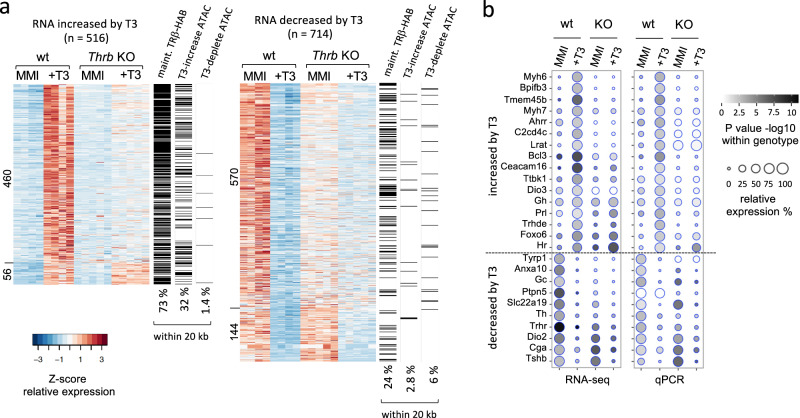
Blunted pituitary gene expression in TRβ-deficiency. **a** Heatmap of T3-induced and T3-suppressed genes detected by RNA-seq (criteria: ≥1.5-fold change in *Thrb*-KO compared to wild type; CPM > 1; *P* < 0.05, Student *t*-test). Groups, 4 or 5 pools of 3 pituitaries of adult male mice in hypothyroid (MMI) or hyperthyroid (T3) conditions. TRβ-deficiency (KO) impairs T3-stimulated changes in most induced (460) and repressed (570) genes. Unimpaired genes in the KO are listed at the bottom (56 and 144 genes). On the *right* of each heatmap, the 3 columns denote presence (black) or absence (white) of specific chromatin sites within 20 kb of the gene body for the following sites: TRβ-HAB maintained binding sites, T3-increased ATAC peaks and T3-depleted ATAC peaks (overall % incidence noted below columns). **b** Dot plots of representative T3-responsive genes determined by RNA-seq and qPCR (qPCR groups, 4 pools of 3 pituitaries). Statistical comparisons within a genotype determined by Student’s t-test.

We determined the requirement for TRβ for pituitary gene expression by transcriptome analysis of *Thrb*-KO and control mice in hypothyroid and hyperthyroid conditions. In wild-type adult male mice, T3-induced 516 genes and suppressed 714 genes (Fig. 5a). The regulation of representative novel and known genes was corroborated by qPCR analysis (dot plots in Fig. 5b). Pituitary hormone genes responded as reported previously^7,8^. T3-induced *Gh* and *Prl* but suppressed *Tshb* and *Cga* subunit genes of TSH. Genes for gonadotropins (*Fshb*, *Lhb*) and Acth (*Pomc*) showed little response (Supplementary Fig. 4).

TRβ-deficiency blunted the response of most genes in both induced and suppressed groups (~89% and ~79% of genes, respectively), indicating that TRβ broadly determines transcriptional sensitivity of the pituitary gland to T3 (Fig. 5a, b). The limited impairment in *Thrb*-KO mice of some T3-responsive genes, such as *Tshb* presumably reflects compensation by the T3 receptor (TRα1) encoded by the *Thra* gene^13,15^.

### Pituitary gene expression associated with T3-regulated chromatin

**Fig. 6:**
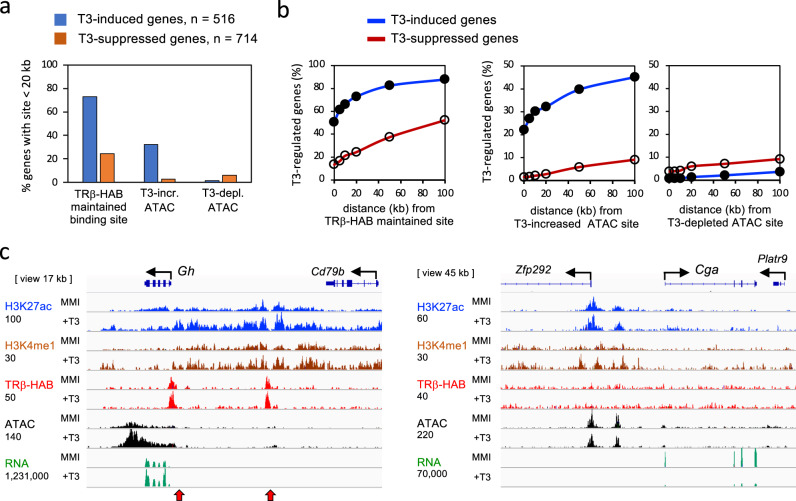
T3-regulated chromatin and pituitary gene expression. **a** Column graphs of the association of T3-dependent pituitary gene expression with TRβ-HAB bound sites (maintained category), T3-increased ATAC sites, and T3-depleted ATAC sites within 20 kb of the gene body. Analysis of 8384 maintained TRβ-HAB sites; 1424 T3-increased ATAC sites and 352 T3-depleted ATAC sites (Fig. 4c). **b** Curve plots of the association of T3-dependent pituitary gene expression with TRβ-bound sites, T3-increased and T3-depleted ATAC sites at distances up to 100 kb from the gene. **c** Example of the T3-induced *Gh* gene showing TRβ-bound sites (red arrowheads), T3-regulated open chromatin (ATAC) and histone marks. In contrast, the strongly T3-suppressed *Cga* gene lacks obvious regulation of chromatin by T3 or detectable TRβ binding. Raw reads scale on *left*, below each mark label.

To support a role for T3-regulated pituitary chromatin sites as enhancers, we investigated the association of T3-dependent gene expression with T3-regulated chromatin sites. TRβ-HAB binding sites were found near a higher proportion of T3-induced genes than T3-suppressed genes (~73% and ~24%, respectively, within 20 kb of a gene, Figs. 5a, 6a). Genome-wide analysis demonstrated this higher incidence (up to 3-fold) of TRβ-bound sites with T3-induced genes over distances up to 100 kb from the gene (Fig. 6b). A substantially higher proportion of T3-increased open chromatin (ATAC) peaks was also detected near T3-induced than T3-suppressed genes (~10-fold higher incidence, within 20 kb of a gene, Figs. 5a,  6a, b). In marked contrast, T3-depleted ATAC peaks were rarely associated with T3-induced or T3-suppressed genes. Thus, T3-induced genes are associated with TRβ binding and T3-increased open chromatin characteristics of putative positive enhancers. However, T3-suppressed genes display a weaker association with TRβ-binding and poor association with chromatin opening or closing, precluding simple generalizations about characteristics of negative enhancers for T3-mediated repression.

As an example, the growth hormone gene (*Gh*), a T3-inducible gene, displays TRβ-bound sites with T3-inducible chromatin opening and extensive histone modifications (Fig. 6c). The TRβ-bound site nearest the gene is consistent with in vitro transactivation studies of the rat *Gh* promoter^1,40^. In contrast, some T3-suppressed genes including the gene for the alpha subunit of TSH (*Cga*) lacked detectable TRβ binding or T3-regulated open chromatin despite the powerful suppression of *Cga* expression by T3 (Fig. 6c).

We demonstrated T3-inducible enhancer activity for representative TRβ-bound sites in transactivation assays using luciferase reporters in transfected cells. Supplementary Fig. 5 demonstrates enhancer activity for binding sites in the *Ceacam16* and *Thrb* genes. We further tested the role of DR4 motifs predicted by the genome-wide motif analysis. Mutagenesis of DR4 motifs in the *Ceacam16* and *Thrb* sites showed that these motifs were essential for T3-inducible activity.

### Inducible chromatin opening at T3-sensitive pituitary control sites

**Fig. 7:**
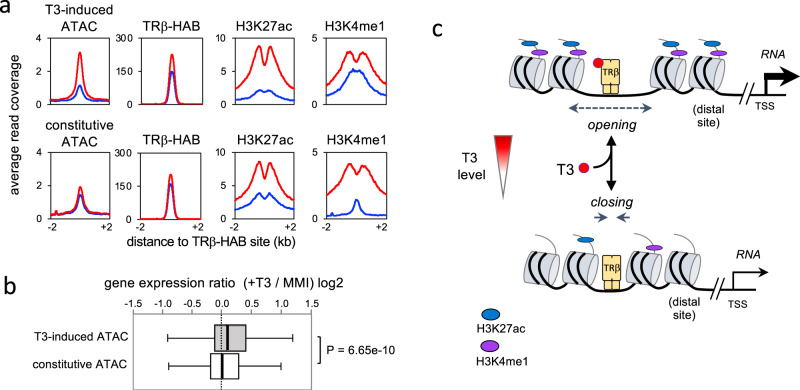
Inducible open chromatin at T3-sensitive sites. **a** Comparison of T3-inducible versus constitutively open chromatin (ATAC peaks) at T3-sensitive, putative enhancer sites in the pituitary gland. Analysis of 1,008 inducible ATAC sites versus the top 1008 constitutive ATAC peaks (of 5809 non-regulated ATAC peaks). Both groups maintain TRβ binding regardless of T3 status and have DR4 as the top motif. **b** Box plot of distribution of T3-regulated genes (RNA level) for both groups of ATAC sites. The median (line in 50% range box) differs significantly. Genome-wide analysis of all genes within 100 kb of ATAC sites (2,030 genes for T3-increased ATAC peaks; 2808 genes for constitutive ATAC peaks). Statistical analysis by Mann–Whitney test. **c** Simplified model for a T3-activated pituitary enhancer. Receptor-bound enhancers, typically at distal sites, dynamically and reversibly control chromatin opening and histone modifications according to the T3 level.

Our findings identify T3-inducible chromatin opening at a specific subset of TRβ-bound chromatin sites suggesting a key property of certain putative T3-sensitive enhancers in the pituitary gland. Of sites with maintained TRβ binding, ~15% (1008/6817) have T3-inducible chromatin opening (ATAC peaks, see Fig. 4d) whereas ~85% have constitutively open chromatin. We tested the hypothesis that T3-regulated gene expression is associated with inducible chromatin opening at putative enhancers by comparing equal groups of T3-inducible versus constitutively open sites (Fig. 7a). Both groups display comparable TRβ-HAB binding with moderate increases in average read counts in response to T3 (~76% and 57% increases in peaks, respectively). Both groups also display T3-induced histone modifications (H3K27ac, H3K4me1) and contain DR4 as the top motif (*P* < 1e−607 and <1e−533, respectively). However, genome-wide analysis showed a stronger association of T3-activated genes with inducible rather than constitutively open ATAC peaks (Fig. 7b), supporting a role for T3-inducible chromatin opening in pituitary gene activation (Fig. 7c).

## Discussion

We find that TRβ binding sites in pituitary chromatin are determined at both tissue- and gene-specific levels. Tissue-specific determinants substantially dictate genomic binding sites as indicated by evidence that of pituitary binding sites, only ~12% are shared with cerebral cortex (Fig. 2) and ~15% are shared with retinal tissue^43^. Tissue-specific determinants may include cell type-specific transcription factors that stimulate chromatin opening or otherwise facilitate T3 receptor binding^2^. In the pituitary, the *Gh* gene is highly induced by powerful pituitary cell lineage activators such as Pit1, which may initiate activation and chromatin opening^1,44^. Although the T3 receptor may be a later-stage regulator, it mediates critical adjustable control over levels of *Gh* expression in response to T3. We find that T3 induces extensive chromatin remodeling and histone acetylation around the *Gh* locus potentially involving an interplay of T3-regulated enhancers and complex upstream control regions^45^. The findings suggest that impaired chromatin regulation contributes to GH-deficiency in hypothyroidism^3,5^.

A second level of specificity resides within the pituitary genome itself as TRβ binding sites are not equally responsive to T3. T3 induces chromatin opening and histone modifications primarily at sites that maintain TRβ binding regardless of T3 status. These sites represent the predominant category of receptor-occupied site in untreated conditions with physiological T3 concentrations. In contrast, sites at which binding is determined by T3, that is de novo induced or abolished, show comparatively little modification of chromatin by T3. We speculate that persistent receptor-enhancer interactions, perhaps aided by a high incidence of near-optimal DR4 sequence motifs, create a poised state capable of dynamic responses to T3 fluctuations (Fig. 7c). The moderate, relative increase in TRβ binding at some of these sites might reflect further stabilization by T3-induced conformational changes as the enhancer shifts to a more active state.

The in vivo tissue data available suggest that shifts in receptor binding are a general response to T3 although the patterns vary. In pituitary tissue, the maintained sites represent the majority of occupied sites when T3 is in physiological ranges. Potentially differing from our findings, a study of the liver reported that most receptor binding reflects T3-induced de novo recruitment based on chromatin immunoprecipitation with an antibody against T3 receptors encoded by both *Thrb* and *Thra* genes (i.e., TRβ and TRα1)^31^. However, another liver study using a knockin tag on TRβ1 reported that ligand increases the degree of binding rather than de novo binding at many sites^29^. An immunoprecipitation study of a human thyroid cell line in culture suggests that most receptor binding reflects T3-stimulated occupancy^46^. However, differences in experimental design, bioinformatics criteria, and technical detection of low levels of receptor binding preclude generalized conclusions between studies at the present time.

Most T3-dependent pituitary genes display some degree of resistance to T3 in *Thrb* KO mice indicating that TRβ mediates transcriptional sensitivity. The full phenotype is probably partly masked by TRα1 substituting for TRβ^13,15^. Current views of pituitary responses to T3 often focus on GH and TSH as clinically relevant, endpoint hormones in the circulation^5,9^ but our identification of several hundred T3-responsive genes suggests a broader spectrum of primary transcriptional actions at the genomic level.

For T3-induced genes, our results support a model of direct activation and chromatin opening at TRβ-bound sites (see later discussion). However, for T3-suppressed genes, low associations with T3-regulated open chromatin or TRβ binding suggest less obvious means of control. Mechanisms of repression by T3 present a long-standing puzzle. Hypothetical negative enhancers might be expected to display chromatin closing at receptor-bound sites commensurate with the extent of gene suppression by T3. However, some potently suppressed pituitary genes such as *Tshb*, *Cga* (TSH subunits), and *Trhr* (TRH receptor) (Fig. 5b, Supplementary Fig. 4), surprisingly lack T3-regulated open chromatin or detectable TRβ binding. These findings may support long distance or indirect modes of suppression^47,48^ and challenge earlier proposals of direct repression of *Tshb* and *Cga* based on transfection assays of short promoter fragments in vitro^49,50^. Although previous studies of short promoter fragments implied that negative enhancers reside close to the transcription start site, at the genome-wide level, our analysis did not reveal obvious enrichment of T3-regulated chromatin sites in the promoter-proximal region of T3-suppressed genes (Fig. 6b). Our in vivo evidence raises the possibility that T3-mediated pituitary gene suppression involves specialized forms of genomic control not involving obvious changes in chromatin accessibility or commonly studied histone modifications. Future studies might investigate other types of histone modifications, including active or repressive marks. There may be varied means of negative regulation. A rare example of a negatively regulated gene that displays some, modest chromatin closing at a receptor-bound site is *Opn1sw* (S opsin) in the retina^43^. A liver study using tagged TRβ1 suggested that binding sites associate with both induced and repressed genes but with shifting cofactor interactions in each case^29^. A functional role for any site in mediating gene repression by T3 in vivo remains to be demonstrated.

We propose a model for pituitary gene activation in which poised receptor-enhancer complexes adapt dynamically to T3 levels (Fig. 7c). This model could explain the sensitivity of the pituitary to T3 since persistently bound, poised receptors could confer immediacy of response to T3 fluctuations. Our findings are compatible with views that T3 stimulates histone acetylation^25,26,28,29,51^ but also implicate opening of the nucleosome array at poised sites in pituitary chromatin. It is unclear if TRβ can bind to a pituitary enhancer in a fully closed chromatin state. Limited, pre-existing opening, perhaps primed by cell-specific factors (Fig. 2) may allow access for TRβ regardless of T3 levels. Rising T3 levels may stimulate TRβ-bound sites to open further by recruitment of remodeling factors that displace nucleosomes^33^, allowing association with mediator and activation complexes^27^. In vitro studies suggest that Brahma-related gene-1^52,53^ and other SWI/SNF remodeling factors^46^ can modify T3 receptor activity in cells in culture. A key feature of a poised receptor model is its reversibility by declining T3 levels which would close chromatin, deacetylate histones and reduce gene expression in hypothyroidism. This proposal focuses on receptor-bound enhancers with T3-inducible open chromatin but does not exclude a role for other categories of binding sites.

Future studies might test the hypothesis that in the pituitary, T3 shifts the association of receptor-enhancer complexes with histone modifying or remodeling factors. Studies of a thyroid cell line in culture suggest that T3 modifies interactions with SWI/SNF factors^46^ and in the liver suggest that T3 modifies genome-wide cofactor associations in a coregulator shift model^29^. However, the in vivo picture is far from complete because of limited in vivo tissue data and a lack of comparable analyses between available studies. We anticipate that there is not a single, uniform type of T3-regulated enhancer and that tissue-specific specializations may reflect functional adaptations for each tissue.

Knockin tags are increasingly used as a powerful tool to isolate transcription factors and bypass a lack of specific reagents for chromatin-binding studies. Any tag may disturb the function of the gene, or the activity or stability of the product, even if only subtly, which is difficult to exclude entirely. For this reason, we employed an independent screen for enhancers by analysis of T3-regulated open chromatin, providing mutual support for findings obtained using the TRβ-HAB model. Mutations within the TRβ C-terminus as occur in human resistance to thyroid hormone disrupt T3-dependent transactivation^54,55^. However, this is not the case with the HAB tag which extends beyond but does not change the C-terminus or the AF2 activation domain of TRβ. Interestingly, variable amino acid residues that extend beyond the AF2 domain are not conserved between nuclear receptors^56,57^ and form a disordered extension in a crystal structure of TRα1^57^, which may explain why the HAB tag does not inhibit transactivation. The similar T3-sensitive transactivation by tagged or non-tagged receptors (Supplementary Fig. 1) and lack of overt phenotypes in *Thrb*^HAB/HAB^ mice suggest that any alterations in vivo would be minor. The utility of the TRβ-HAB model is supported by the consistency of chromatin binding sites identified in the liver using an N-terminal HA-TRβ1 tag^29^ or the C-terminal TRβ-HAB tag (Supplementary Fig. 6). Thus, tag location and different pull-down methods (immunoprecipitation or affinity-purification) do not overtly distort outcomes. Further support is provided by the finding of concordant sites in lipogenic genes in the liver using either virally expressed TRβ1 with an N-terminal biotinylation tag or the TRβ-HAB model^58^.

Overall, our findings of dynamic control of pituitary chromatin by T3 suggest a genomic basis for understanding pituitary function and dysfunction as well as responses of the pituitary to widely used treatments for hyperthyroidism or hypothyroidism^9^.

## Methods

### Mouse genetic models

The *Thrb*^HAB^ allele expresses TRβ proteins (TRβ1 and TRβ2) fused to a peptide with a hemagglutinin (HAx2) tag and a site for biotinylation by prokaryotic BirA ligase, modified from a published tag^30^. The tag was inserted at the endogenous *Thrb* gene by homologous recombination in W9.5 (129/Sv) embryonic stem cells^14^. The construct included a self-excising ACN neomycin-resistance cassette^59^, a 3.6 kb 5’ homology arm, and 4.6 kb 3’ homology arm with coordinates relative to the ATG start of the *Thrb* b2 exon: +47,877 to +51,787 and +51,791 to +56,830, respectively. Targeting was confirmed by Southern blot and sequencing analyses. Germline transmission was established by crossing with C57BL/6J mice (Jackson Lab # 000664). PCR genotyping was performed using 3 primers as follows: TRbF, 5’-CCA TGT GAC ACA CTT TTG GC-3’; TRb-R, 5’-GTG CTG CAG GAA TGA CAA GA-3’; HAB-R, 5’-CA TTA CTC GTG CCA CTC GAT CTT C-3’ giving bands of 186 and 321 bp for wild-type and HAB alleles, respectively, using conditions: denaturation at 94 °C for 2 min, 33 cycles of 94 °C for 30 s, annealing at 61 °C for 30 s, extension at 72 °C for 40 s, then extension at 72 °C for 5 min. Genotyping primers and other primers used in this study are listed in Supplementary Table 3.

To biotinylate receptors, *Thrb*^HAB^ mice (129/SvJ × C57BL/6J background) were crossed with *Rosa26*^BirA^ mice (background of 129/OlaHsd backcrossed at least 3 generations onto FVB/N, as described by the supplier; Jackson Lab #010920)^35^. Genotyping was performed using PCR protocols provided by the Jackson Lab. Anterior pituitary-specific nuclei were labeled by crossing *Rosa26*^*Sun1-GFP*^ mice (*Gt[ROSA]26Sor*^tm5(CAG-Sun1/sfGFP)Nat^, Jackson Lab #030952)^42^ and *Thrb*^*b2Cre*^ mice^43^ on a C57BL/6J × 129/Sv background. *Thrb*^-/-^ mice were on a C57BL/6J background (Jackson Lab # 003462)^14^. *Thrb*^b1-lacZ^ and *Thrb*^b2-lacZ^ mice carry knockin reporters at the endogenous *Thrb* gene (backcrossed for several generations onto C57BL/6J) as described^17^.

Unless otherwise noted, adult male groups (2–4 months old) were analyzed. Mice were kept in a 12 h light/12 h dark cycle and tissues taken in the light phase between ~14.00 and 18.00 h. To induce hypothyroidism, 0.05% Methimazole (MMI) and 1% potassium perchlorate (KClO_4_) were added to the drinking water for 4 or 5 weeks. Subgroups were made hyperthyroid by co-administration of 0.5 μg/ml T3 in the drinking water for the final week^60^. Studies were performed in accordance with the NIH Guide for Care and Use of Laboratory Animals and protocols approved by NIDDK Animal Care and Use Committee.

### Tissue immunostaining and histomorphometry

Adult male pituitary glands were fixed in 2% paraformaldehyde (PFA) and 12 μm cryosections prepared for staining using established histological methods^17^. Antibodies are listed in Supplementary Table 4. Images were captured on a Leica SPE2 confocal microscope and processed using ImageJ. Pituitary glands were sectioned in the coronal plane and areas of a half-lobe of the anterior pituitary after immunostaining for GH were measured using ImageJ. For thyroid histology, the entire gland attached to the trachea was fixed in 2% PFA/3% glutaraldehyde for 2 days, treated in 0.1 M EDTA for 1 week, then embedded in methacrylate plastic. To retain morphology, both lobes were sectioned in place on the trachea at 4 μm thickness in the transverse plane on a microtome, then stained in hematoxylin and eosin^17^. Areas of colloid within follicles (20 views) and of total lobes (10 views) were measured in transverse, mid-lobe sections using ImageJ.

### Auditory measurements

Auditory-evoked brainstem responses were tested using established procedures on adult mice under avertin anesthesia^17^.

### Hormone measurements

TSH, total T4 and total T3 levels in serum samples were measured using Milliplex magnetic bead panels. T4 and T3 were measured with a rat panel (RTHMAG-30K; MilliporeSigma)^61^ according to the manufacturer’s instructions. Independent analyses of T4 and T3 in untreated groups by LC-MS/MS, as described^62^ gave similar outcomes. TSH and GH were measured using a Milliplex MAP Mouse Pituitary Magnetic Bead Panel Mouse (MPTMAG-49K; MilliporeSigma) and Millipore Luminex200 plate reader with Millipore Analyst Software (MilliporeSigma)^63^.

### Transfection and luciferase assays

TRβ2 or TRβ1 mouse cDNAs with or without fusion to a 3’ HAB tag were inserted into pcDNA3 expression vectors. HEK293T cells were cultured in Dulbecco’s modified eagle medium (DMEM) with 10% fetal bovine serum (FBS). Subconfluent cells in 12-well plates were transfected with 100 ng of a T3-responsive luciferase reporter, 10 ng phRG-tk renilla control plasmid and 200 ng of receptor expression vector with or without 5 ng of a BirA expression plasmid^30^, using GenJet^TM^ reagent (SignaGen Lab). A BirA expression Luciferase reporter plasmids with a tk promoter carried the following response elements: TREpal0^64^ in a pGL2 vector containing 2 copies of a palindromic repeat (5’-AGG TCA TGA CCT GAG ATC TCA GGT CAT GAC CT-3’) and DR4 in a pGL4 vector containing 2 copies of a direct repeat with 4 base spacer (5’-AGG TCA CTT CAG GTC ATC ACG TAA CTG ATG TAG GTC ACT TCA GGT CA-3’). Charcoal-stripped FBS was used with T3 added at stated final concentrations the day after transfection. Luciferase activity was measured 24 h after T3 treatment.

GH3 rat pituitary cells were cultured in DMEM with 10% FBS and transfected using GenJet^TM^ reagent (SignaGen Lab). The next day, cells were treated with or without 10 nM T3 in DMEM with 2.5%, charcoal-stripped FBS. One day later, cells were harvested and luciferase activity measured on a GloMax 96 Microplate Luminometer, after adding Dual-Glo Luciferase reagent, then again after adding Stop & Glo reagent (Promega). Genomic DNA fragments containing TRβ-binding sites were cloned into chromatin-forming pREP4-Luc2 reporter vector. Luciferase activity was calculated as the ratio of firefly luciferase (Luc2)/renilla control activity. Samples were analyzed in triplicate and experiments repeated at least twice.

### Cytoplasmic/nuclear fractionation of transfected cells and western blot analysis

Cell pellets were obtained from 10 cm plates of HEK293T cells transfected with 5 μg of pcDNA3-TRβ1, pcDNA3-TRβ2, pcDNA3-TRβ1-HAB or pcDNA3-TRβ2-HAB using GenJet^TM^ reagent (SignaGen Lab). Pellets were homogenized in 1 ml of ice-cold cytoplasmic extraction buffer containing 10 mM HEPES, 10 mM KCl, 0.1 M EDTA, 0.1 mM EGTA, 1 mM DTT, 0.5 mM PMSF and protease inhibitor cocktail (Roche 11836145001), incubated on ice for 15 min, then mixed (vortex for 10 s) with 2.5 μl of 20% NP40. After centrifugation at 13,000 × *g* for 30 s, the supernatant (cytoplasmic fraction) was taken and stored at −80C. The nuclear pellet was resuspended by pipetting in 150 μl of nuclear extraction buffer (20 mM HEPES, 0.4 M NaCl, 1 mM EDTA, 1 mM EGTA, 1 m MDTT, 1 mM PMSF and protease inhibitor cocktail). The suspension was vortexed vigorously every few minutes while keeping on ice for 15 min. After centrifugation at 13,000 × *g* for 5 min, the supernatant (nuclear extract) was taken and stored at −80C. Cytoplasmic and nuclear fractions were analyzed by 10% SDS-polyacrylamide gel electrophoresis in MOPS buffer with electro-transfer to PVDF membranes. Rabbit antiserum against TRβ1 (1:2500) or TRβ2 (1:2500)^65^ was used to detect tagged and untagged receptors. The secondary antibody was horseradish peroxidase-goat anti-rabbit IgG (Cell Signaling Technology, 5127) (1:2000). Western blot immunostaining for tubulin and lamin A/C was used to detect cytoplasmic and nuclear markers, respectively, and for actin to indicate protein loading. Secondary antibody was horseradish peroxidase-goat anti-mouse IgG (Cell Signaling Technology, 58802) (1:2000) with detection using Clarity™ Western ECL Substrate (Bio-Rad, Cat 170–5060) and ChemiDoc™ Touch Imaging System (Bio-Rad). Original scans of western blots with markers are included in Supplementary Fig. 7.

### Affinity-purification of biotinylated protein

Nuclear protein extracts were prepared^65^ from pituitaries (pools of 3) of the indicated genotypes (Supplementary Fig. 3) then incubated with streptavidin T1 Dynabeads (Invitrogen, Cat 65601) at 4 °C overnight to bind biotinylated proteins. Beads were washed 3x in 50 mM Tris-HCl, pH 7.5, 400 mM NaCl, 1% Triton X100, 1% Sodium Deoxycholate, 2% SDS, 2 mM EDTA, 0.5 μM PMSF and 0.1% protease inhibitor cocktail. Affinity-purified protein was resuspended in 2x SDS gel loading solution (Quality Biological Inc, #351082661) and eluted from beads by boiling for 10 min. Nuclear extract (input), supernatant (wash) and affinity-purified proteins were analyzed by 10% SDS-polyacrylamide gel electrophoresis in Tris-glycine buffer with electro-transfer to nitrocellulose membrane. Rabbit antiserum against TRβ2 (1:2500)^65^ was used to detect tagged and untagged receptors. Secondary antibody was horseradish peroxidase-goat anti-rabbit IgG (ThermoScientific G21234)(1:10,000). Signal was detected using Pierce ECL Plus Western Blotting Substrate (Thermo Scientific, Cat 32106) and Kodak Biomax film exposure.

### Quantitative PCR (qPCR) analysis

Groups contained 4 replicates, each representing a single pituitary or ≥2 pooled pituitaries (male). Random hexamer primers were used to synthesize cDNA from total RNA using SuperScript III reverse transcriptase (Life Technologies). First-strand cDNA was mixed with 250 nM of test gene primers with Power SYBR master mix in a StepOne Plus real-time PCR system (Life technologies). Relative RNA levels were normalized to *Actb* as a reference gene. See Supplementary Table 3 for primer sequences.

### Statistics and reproducibility

Data are shown as mean ± SD unless otherwise noted. Pairwise comparisons were performed using Student’s t-test. Multiple group comparisons were performed by ANOVA followed where appropriate by posthoc analysis with Tukey’s test for individual comparisons. *P* < 0.05 was considered to indicate significance. A Mann–Whitney test was used in Fig. 7b (https://astatsa.com/WilcoxonTest/). Tests and groups are noted in the figure legends. Statistical tests were performed using GraphPad Prism version 9.2.0.

### RNA-sequencing

Total RNA was prepared from pools of 3 frozen pituitaries per library, using TRIzol Reagent (Invitrogen, Cat 15596-026), then mRNA enriched using Dynabeads mRNA purification Kit (Ambion Cat# 61006) and quantified using Qubit RNA Broad-range assay Kit (Thermo Fischer Scientific). Five μg of total RNA was denatured at 65 °C for 5 min, then incubated with oligo(dT)-beads with rotation at room temp for 5 min. Beads were washed twice in 10 mM Tris-HCl pH7.5, 0.15 M LiCl, and 1 mM EDTA, then mRNA eluted in 10 mM Tris-HCl pH 7.5 at 80 °C for 2 min. Libraries were synthesized using SuperScript Double-Stranded cDNA Synthesis Kit (Invitrogen) and sequencing libraries generated using Illumina Truseq (Cat# 15034288) or ThruPLEX DNA-seq (Takara, R400428) kits. Samples were multiplex-sequenced on an Illumina HiSeq 2500 instrument at the NIDDK Genomics facility. Single-end 50 base reads were mapped to RefSeq mouse transcript database mm9 (Build 37) using BBMap version 36.02 with aligned data in bam file format. Transcripts with >1 counts per million (CPM) in all RNA-seq samples were selected for further analysis.

### ChIP-seq

Pools of 12 frozen pituitaries were cross-linked in 1% formaldehyde in 1x PBS and cOmplete^TM^ protease inhibitor (Roche #11836145001) for 10 min at room temperature, then glycine added at 125 mM final concentration to quench the reaction. Samples were centrifuged at 1400 × *g* for 2 min at room temperature. The pellet was washed by vortexing in 10 mL ice-cold PBS with protease inhibitor, pelleted again, suspended in 2 mL ice-cold cell lysis buffer and homogenized using 40 plunges with pestle A in a 1 mL glass homogenizer on ice. The sample (~2 mL) was transferred into a 15 mL tube. The homogenizer and pestle were rinsed in 2 × 4 mL cell lysis buffer to collect residues. The total 10 mL sample was incubated on ice for 15 min, then centrifuged at 3800 × *g*, for 10 min at 4 °C. The nuclear pellet was resuspended in 1.5 mL nuclear lysis buffer, incubated at room temp for 10 min, then mixed with an additional 1.5 mL of buffer. The sample was sonicated in a 15 mL conical tube (Fisher Scientific sonicator, model FB705) then centrifuged at 18,000 × *g* for 10 min at 4 °C, to yield supernatant (sample) for analysis.

For ChIP-seq assays of histone marks, 3 mL of supernatant was incubated with specific antibody on a rotator at 4 °C overnight, then mixed with 20 μl pre-washed Magna ChIP Protein A + G beads (Millipore, #16-663) for 2 h. Beads were washed in dilution buffer (16.7 mM Tris-Cl, pH 8.0, 167 mM NaCl, 1.2 mM EDTA, 1.1% Triton X100), then in low-salt (20 mM Tris-Cl, pH 8.0, 150 mM NaCl, 2 mM EDTA, 1% Triton X100, 0.1% SDS), then high-salt (20 mM Tris-Cl, pH 8.0, 500 mM NaCl, 2 mM EDTA, 1% Triton X100, 0.1% SDS), then ice-cold LiCl buffer (50 mM Tris-Cl, pH 7.5, 250 mM LiCl, 0.5% NP-40, 0.5% sodium deoxycholate), then rinsed twice in 1 ml TE buffer (10 mM Tris-Cl, pH 8.0, 1 mM EDTA). Proteins were eluated from beads in 250 μl of buffer (1% SDS, 0.1 M NaHCO3), then cross-links reversed by addition of 10 μl 5 M NaCl and incubation at 65 °C for 4 h. To release DNA, proteinase K was added at final concentration 0.5 mg/mL with 5 μL of 0.5 M EDTA and 10 μL of 1 M Tris pH 6.5, with incubation at 56 °C for 1 h. DNA was collected using a magnet rack, purified by QIAquick PCR Purification Kit (QIAGEN, 28104) and quantified using Qubit dsDNA HS Assay Kit (Thermo Fisher Scientific, #Q32851). ChIP-seq libraries were generated using an Illumina Truseq kit (Cat# 15034288).

### ChAP-seq

For ChAP-seq, pools of 12 pituitaries (*Thrb*^HAB/HAB^;*Rosa26*^BirA/BirA^ and control Rosa26^BirA/BirA^ genotypes, males) were processed as for ChIP-seq to the supernatant sample stage. The ChAP-seq method was modified from published protocols^66^. A 3 mL sample was incubated with pre-washed Streptavidin T1 beads (Invitrogen Dynabeads, #65601) on a rotator at 4 °C overnight. All subsequent steps were as in the ChIP-seq protocol. ChAP-seq libraries were generated using Illumina Truseq kit (Cat# 15034288).

### ATAC-seq library construction and sequencing

Nuclei were isolated from single pituitaries or pools of 2 pituitaries, frozen, from hypothyroid or hyperthyroid *Thrb*^b2Cre/+^;*Rosa26*^*S*un1-GFP/Sun1-GFP^ male mice. Tissue was homogenized in 0.5 mL ice-cold nuclei isolation buffer (NIB) (20 mM Tris-HCl, 50 mM EDTA, 5 mM Spermidine, 0.15 mM Spermine, 0.1% mercaptoethanol, 40% Glycerol, pH 7.5) with EDTA-free protease inhibitor (Roche 11836170001) in a 1 mL glass homogenizer using pestle A. The homogenate was filtered through a cell strainer (Corning 352235 USA) and centrifuged at 500 × *g* for 5 min at 4 C. The nuclear pellet was washed with resuspension buffer (RSB) (10 mM Tris-HCl, 10 mM NaCl, 3 mM MgCl2, pH 7.4) containing 0.4% IGEPAL^®^CA-630 (Sigma-Aldrich, I8896). The pellet was resuspended in 10 mL RSB with 0.4% IGEPAL-630, incubated with 10 μg of anti-GFP antibody (Abcam ab290, RRID: AB_303395) for 30 min, then incubated with 60 μL of Dynabeads for 20 min. Bead-bound nuclei were passed through a 20 μm strainer (Partec 04-0042-2315) and washed in 3 × 5 mL, 2 × 1 mL, and 1 × 1 mL RSB with 0.4% IGEPAL-630. All steps were performed on ice with incubations using an end-to-end rotator. To calculate the concentration of nuclei, a 5% sample of bead-bound nuclei was sonicated, then dsDNA concentration measured using a mouse genomic DNA standard curve. Approximately 50,000 bead-bound nuclei were incubated in a 50 μL volume containing 25 μL of 2X Tagment DNA buffer, 2.5 μL Tn5 transposase, 0.5 μL of 1% digitonin, and 22 μL nuclease-free water (Illumina FC-121-1030; G9441, Promega) for 30 min at 37 °C. Fragmented genomic DNA was recovered using MinElute spin columns (Qiagen #28604) and amplified by five cycles of qPCR. A 10% volume of the PCR mixture was subjected to an additional 20 cycles of SYBR green-based qPCR while the remaining sample was kept on ice. The qPCR data suggested the number of additional cycles required to generate product at 25% saturation. Typically, 4–7 PCR cycles were added to the initial 5 cycles. Amplified DNA was purified on AMPure XP beads (Beckman A63881), analyzed on an Agilent Bioanalyzer and sequenced (50 base, single end) on an Illumina HiSeq 2500 instrument.

### Processing of ATAC-seq data

Sequencing reads were aligned to mm9 genome build using Biotie2. We determined open chromatin peaks using MACS2 (cutoff *P* value = 1e−5, bandwidth = 300 bp; criteria for intersecting peaks in replicates were a minimum 75% overlap and minimum 1 bp common region). Differential analysis was performed using edgeR 3.16.5 with parameters: log2(fold-change) ≥ 0.58 for enrichment, log2(fold-change) ≤ 0.58 for depletion, and adjusted *p*-value threshold = 0.05. Transcription factor binding motifs were obtained from the HOMER database^67^.

### Processing of RNA-seq, ChAP-seq, and ChIP-seq data

Gene annotation was based on the NCBI reference sequence database (RefSeq) for mouse genome assembly mm9. Transcription start site (TSS) regions were defined as ±1 kb from TSS of reference genes and distal regions as all regions except TSS regions. Differential analysis of RNA-seq data was based on ≥1.5-fold change and P < 0.05 using R software. Gene expression heatmaps and dot plots generated from RNA-seq or qPCR data were created using R (version 4.1.1).

For ChIP-seq, regions enriched for histone marks were identified using SICER^68^ with a 200 bp window and estimated false discovery rate (FDR) threshold of 1e−3. For ChAP-seq analyses of TRβ-HAB data, peaks were called using SICER based on a window of 50 bp and FDR threshold of 1e−6 by differential analysis of *Thrb*^HAB/HAB^;*Rosa26*^BirA/BirA^ (HAB;BirA) and control *Rosa26*^BirA/BirA^ (BirA) genotypes. We identified pituitary T3-induced peaks, T3-abolished peaks, and maintained peaks, by comparison of 14,763 HAB peaks in hyperthyroid (+T3) conditions with 17,031 HAB peaks in hypothyroid (MMI) conditions. Identified peaks (FDR 1e−6; 50 bp windows) were assigned with respect to response to T3, as follows: maintained sites were defined as peaks found in both MMI and +T3 conditions with at least 1 bp overlap between peaks in each condition; abolished and de novo categories represented remaining peaks in the MMI and +T3 conditions. Genomic heatmaps were generated with 50 bp resolution and ranked according to the intensity at the center of a ±4 kb window. Average profile curves represent number of reads for each group with the peak at the center of a ±2 kb window. IGV gene maps were created using Integrative Gene Viewer version 2.8.2. Gene ontology for TRβ-HAB or ATAC peaks was analyzed using GREAT version 4.0.4 with default parameters (http://great.stanford.edu/public/html/). We assessed the consistency of chromatin binding peaks detected with TRβ-HAB (C-terminal tag, streptavidin-based ChAP) and another knockin, HAx3-TRβ1 (N-terminal tag, antibody-based ChIP)^29^ by analyses of datasets for liver^58^ (GEO access # GSE133110 and GSE159648, respectively). See Supplementary Fig. 6.

A summary of genomic datasets generated in this study is listed in Supplementary Table 5.

### Reporting summary

Further information on research design is available in the Nature Portfolio Reporting Summary linked to this article.

### Supplementary information

Supplementary Information file

Description of Additional Supplementary Files

Supplementary Data

Reporting Summary

## Data Availability

All data that support the findings are available within the manuscript and the Supplementary Information. Numerical source data for the graphs in the manuscript are available in a Supplementary Data file. Genomic datasets generated in this work are available at GEO with accession # GSE197703.
