# Supplementary material for: Thyroid hormone-regulated chromatin landscape and transcriptional sensitivity of the pituitary gland
Source: Commun Biol. 2023 Dec 11;6:1253. doi: 10.1038/s42003-023-05546-y (PMC10713718; doi:10.1038/s42003-023-05546-y)
Supplement: Supplementary file 1 — Supplementary Information file [file 42003_2023_5546_MOESM1_ESM.pdf]

SUPPLEMENTARY INFORMATION

“Thyroid hormone-regulated chromatin landscape and transcriptional sensitivity of the pituitary gland”

Cho, Y.-W., Fu, Y., Huang, C.-C. J., Wu, X., Ng, L., Kelley, K.A., Vella, K.R., Berg, A. H., Hollenberg, A.N. Liu, H., and Forrest, D.

This Supplementary Information file contains:

Supplementary Figures 1 – 7

Supplementary Tables 1 - 5

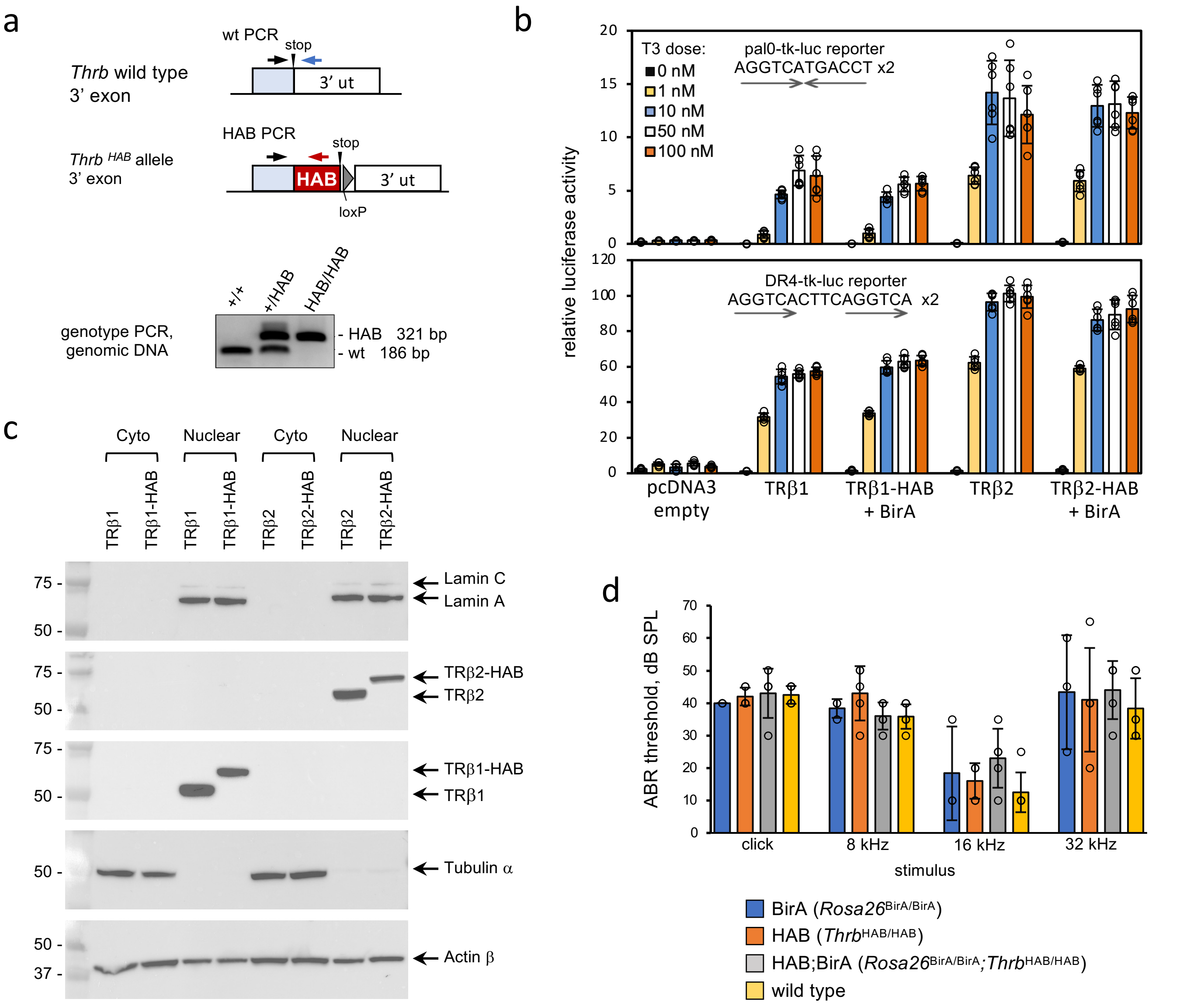

Supplementary Fig 1. *Thrb*^{HAB} knockin allele and tagged receptor function

a, PCR genotyping of *Thrb*^{HAB} allele (arrows denote primers; see Materials and Methods). Gel image shows bands detected for tagged (HAB) and wild type (wt) alleles in mouse genomic DNA.

b, Transactivation of luciferase reporters by tagged and non-tagged receptors in transfected HEK293T cells; pcDNA3, empty vector. Reporters contained two different T3-response elements: pal0, palindromic repeat of AGGTCA; DR4, direct repeat of AGGTCA with 4 base spacer. Each element was present in 2 copies. Experiments were performed in triplicate and repeated twice. Mean ± SD; $P < 0.001$, t-test for each T3 dose (≥1 nM) compared to no T3 added.

c, Detection of tagged and non-tagged receptors in nuclear fractions of 293T cells transfected with receptor expression vectors (noted above lanes, *top*). Nuclear and cytoplasmic fractions of lysates were analyzed by western blot to detect TRβ isoforms and marker proteins noted on the *right*. Lamins (nuclear), tubulin (cytoplasmic), actin (protein loading marker).

d, Auditory-evoked brainstem response (ABR) for 2 month old mice (mixed males and females, n = 5 – 6 except BirA, n = 3). WT, C57BL/6J. Mean ± sem. Normal ABR thresholds were detected for all genotypes ($p > 0.16$; ANOVA, 1-way).

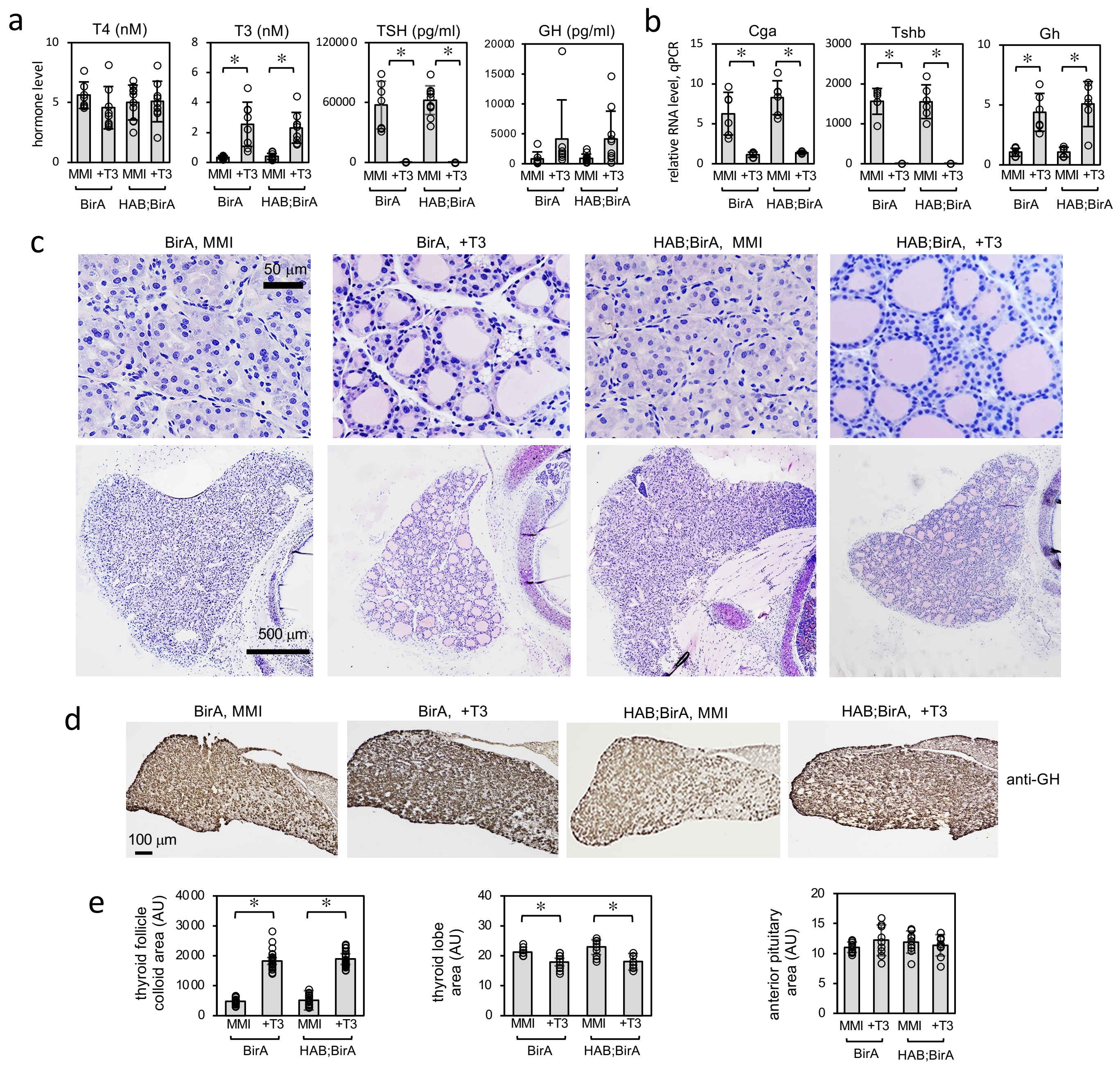

Supplementary Fig 2. Pituitary and thyroid status in hypothyroid and hyperthyroid conditions

a, Serum hormone levels in hypothyroid (MMI) and hyperthyroid (+T3) conditions. Mean ± SD. For both BirA control (n = 7 - 8) and HAB;BirA (n = 9 - 11) adult male mice, MMI in drinking water decreased levels of T4 (~8-fold) and T3 (~2-fold), compared to untreated mice. Addition of T3 increased T3 > 6-fold above MMI-conditions (>3-fold above untreated); T4 remained suppressed. No statistical difference was detected in hormone levels between genotypes. For T3 and TSH, * $P < 0.0001$ for treatment; $P > 0.6$ for genotype (two-way ANOVA and Tukey's posthoc test within genotypes). $P < 0.02$ for GH for treatment; $P > 0.6$ for genotype (two-way ANOVA); Tukey's posthoc test did not reach significance for GH.

b, Pituitary gene expression analyzed by qPCR. Mean ± SD. Responses of Cga, Tshb and Gh, * $P < 0.0001$ for treatment, $P > 0.15$ for genotype (two-way ANOVA and Tukey's posthoc test within genotypes).

c, Thyroid gland histology; 4 µm plastic sections. For both genotypes, MMI enlarged the gland, induced hyperplasia and reduced follicular colloid areas whereas +T3 reverted the morphology to resemble that of untreated mice.

d, Pituitary half-lobe 10 µm cryosections. For both genotypes, MMI and T3 treatments did not change anterior pituitary morphology. Immunostained for GH.

e, Thyroid and anterior pituitary morphology. Thyroid areas, * $P < 0.005$ for treatment, $P > 0.15$ for genotype (two-way ANOVA and Tukey's posthoc test). Anterior pituitary area did not change according to treatment or genotype, $P > 0.6$ (two-way ANOVA); half-lobe areas measured in coronal cryosections using ImageJ (AU, arbitrary units).

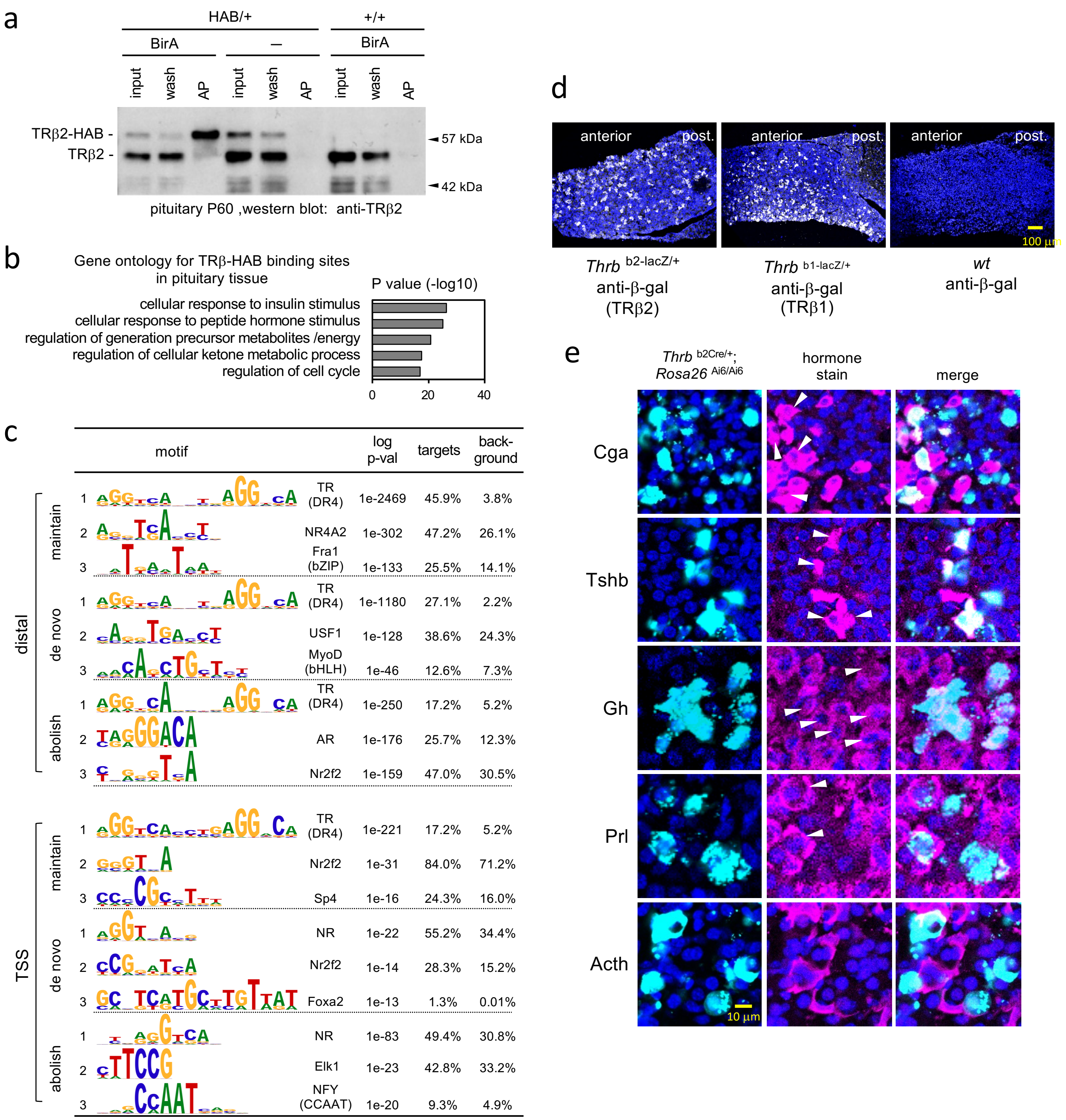

Supplementary Fig 3. ChAP-seq analysis of TRβ binding sites and *Thrb* expression in the anterior pituitary gland

a, Western blot of affinity-purified TRβ-HAB protein (AP lane) in pituitary nuclear extracts from mice carrying *Thrb*^{HAB} and *Rosa26*^{BirA} alleles but not either allele alone. The antibody against the TRβ2 N-terminus detects both tagged and untagged receptors.

b, Gene ontology (GO) analysis of pituitary binding sites detected by ChAP-seq. Genes are listed in Table S5.

c, Motifs in TRβ-HAB site categories with respect to response to T3 (maintain, de novo induced, abolished) in pituitary chromatin identified by HOMER analysis (top 3 motifs).

d, Expression of TRβ2 and TRβ1 isoforms in the anterior pituitary indicated using lacZ knockin reporter alleles for each isoform encoded by the endogenous *Thrb* gene. Immunofluorescence for β-galactosidase protein (white) in cryosections of adult male mice.

e, *Thrb*^{b2Cre} knockin driver expression in the anterior pituitary detected using a *Rosa26*^{Ai6} (ZsGreen) reporter allele. *Thrb*^{b2Cre} expression in anterior pituitary cell populations immunopositive for Cga, Tshb, Gh and Prl (arrowheads). Minimal double-staining detected for Acth-positive cells. Tissue nuclear staining, DAPI (dark blue).

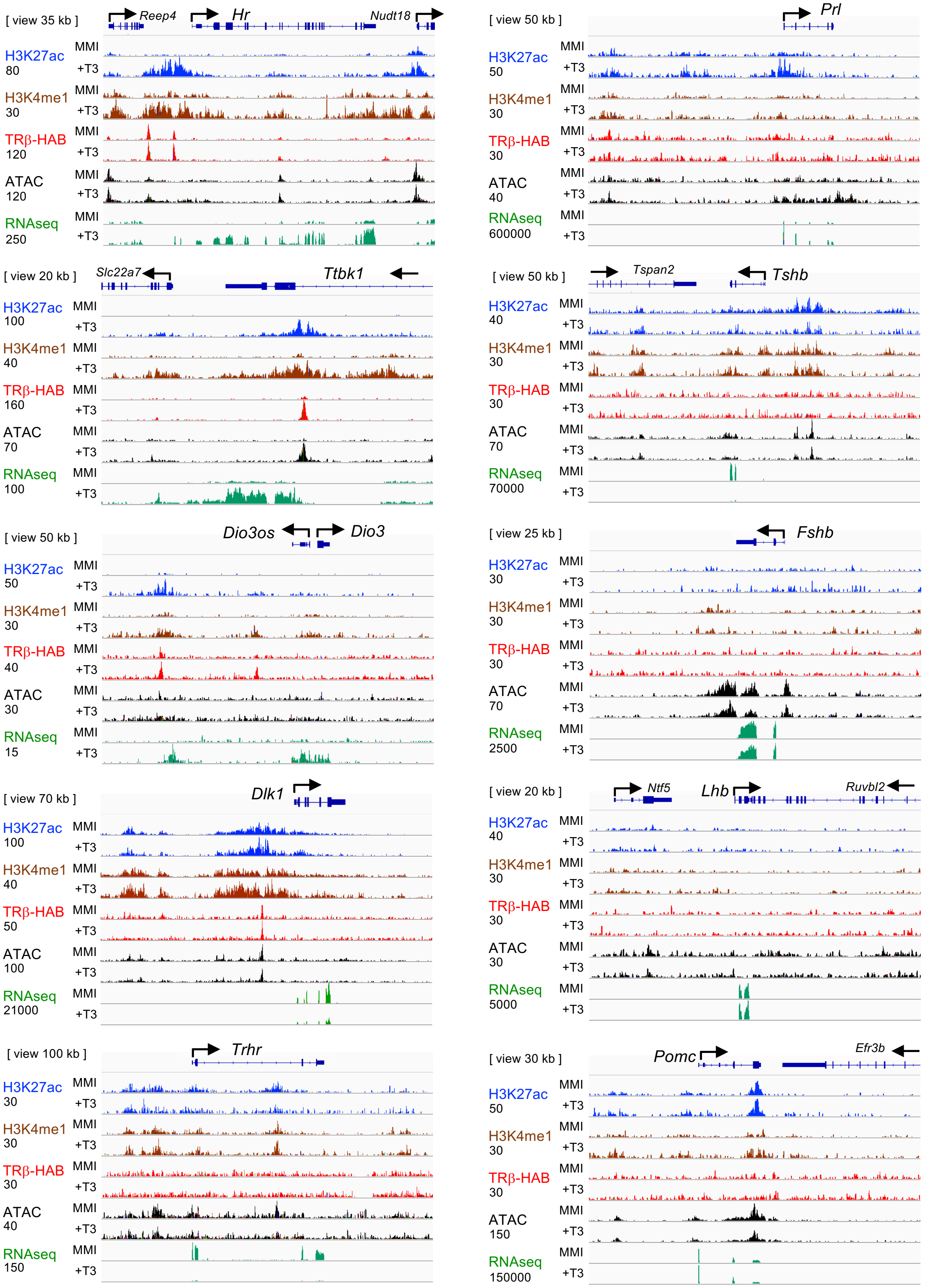

Supplementary Fig 4. Gene maps showing TRβ-binding and chromatin regulation in hypothyroid (MMI) or hyperthyroid (+T3) mice.

Adult male mice. IGV maps; normalized read scales, noted as numbers below each mark.

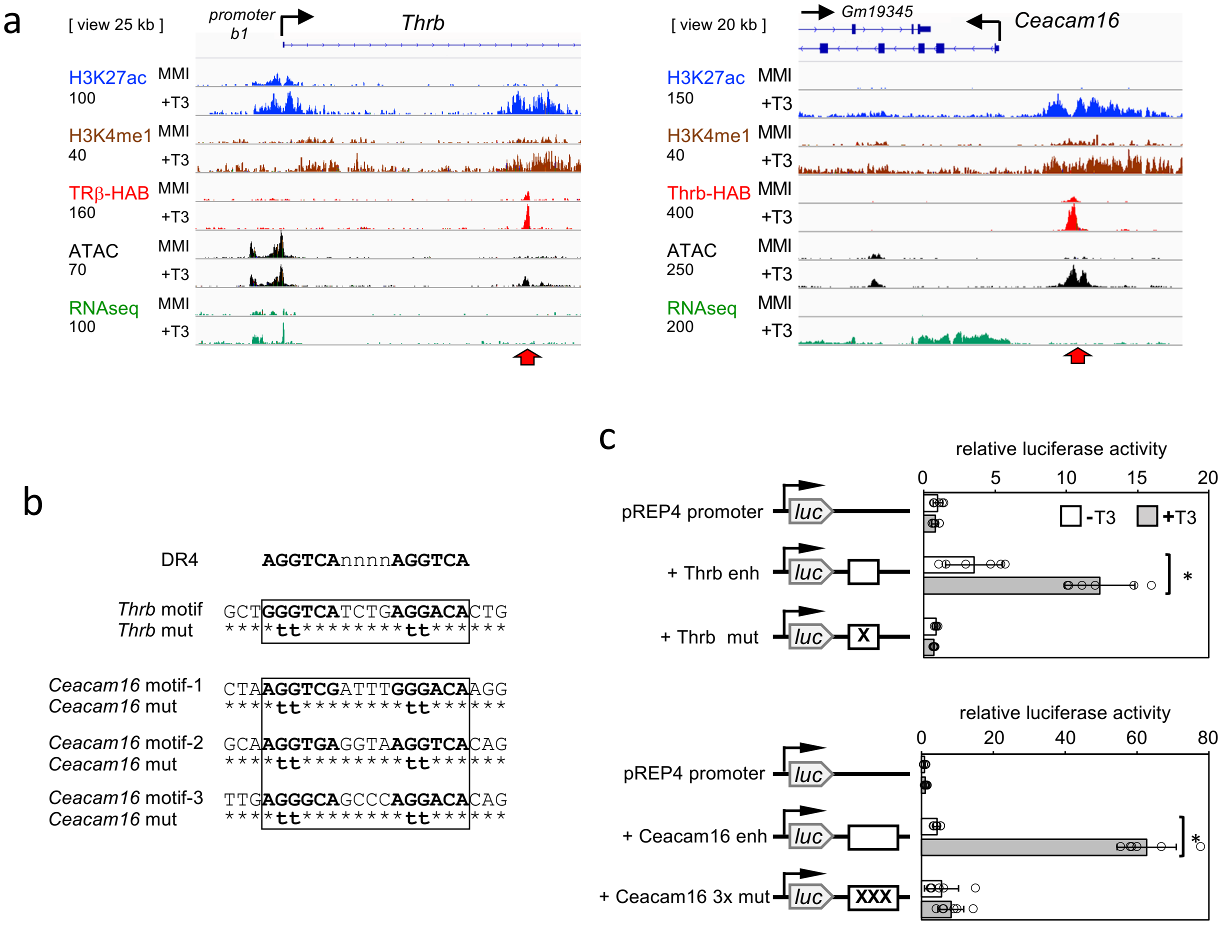

Supplementary Fig 5. T3-dependent enhancer activity of TRβ binding sites

a, T3-regulation at TRβ-bound sites at the *Thrb* and *Ceacam16* loci in pituitary. Each site shows T3-inducible histone marks (H3K4Ac, H3K4me1) and chromatin opening (ATAC). Normalized reads scale, noted below each label on *left*.

b, DR4 motif in the *Thrb* site and triple DR4 motifs in the *Ceacam16* site. Mutations of these motifs (“mut”) abrogated enhancer activity (see panel C).

c, T3-regulated enhancer activity of luciferase reporter constructs in transfected pituitary GH3 cells. Fragments containing binding sites (*Thrb*, 391 bp; *Ceacam16*, 2176 bp) were inserted downstream of a luciferase reporter cassette in a chromatin-forming pREP4 vector. Mean ± SD; *, $p < 0.001$, Student t-test for pairwise comparison of –T3 and +T3 (10 nM) treatments. Experiments performed in triplicate and repeated twice.

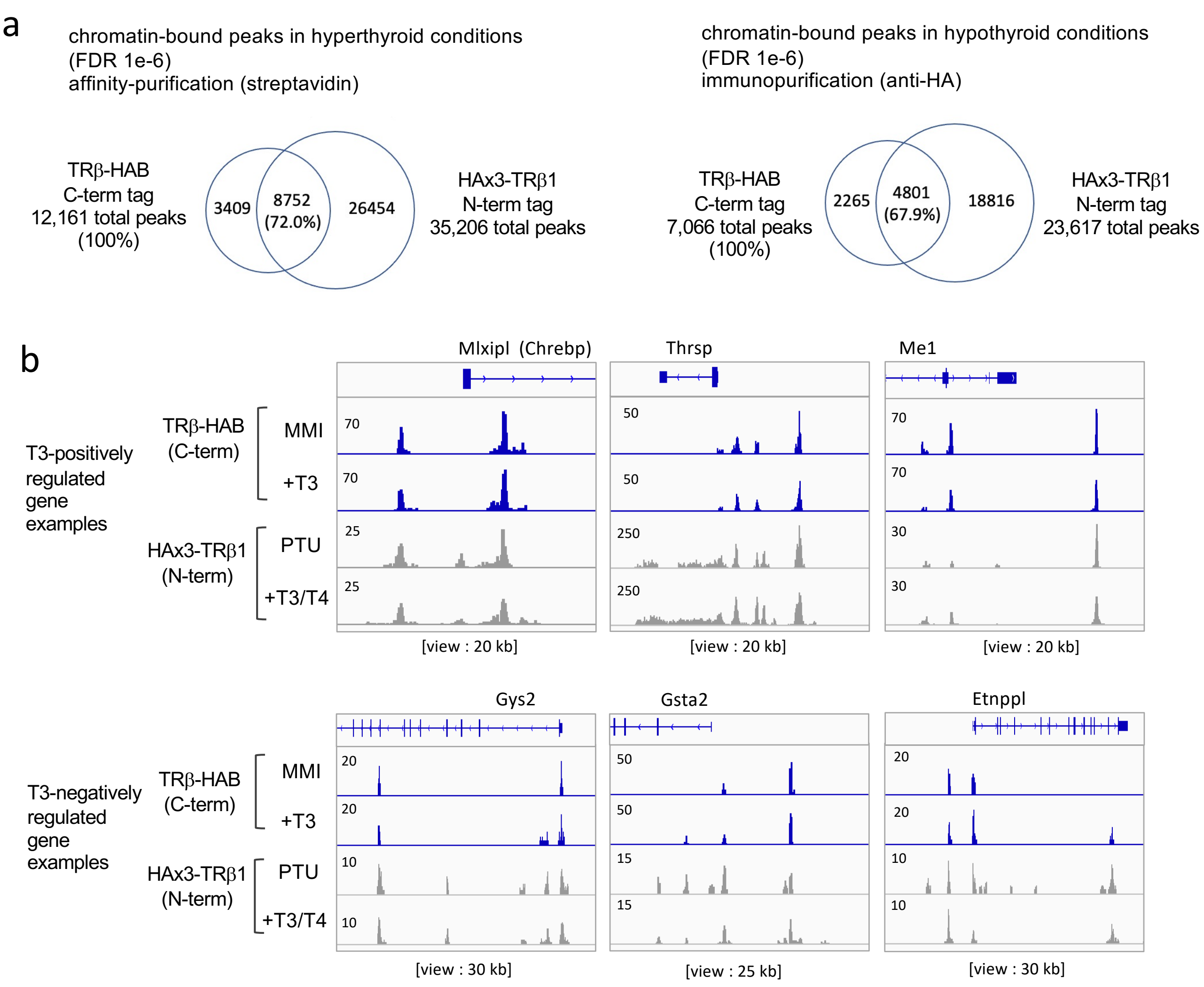

Supplementary Fig 6. Consistency of TRβ-binding peaks in liver chromatin detected with knockin C- or N-terminal tags

a, Venn diagrams of peaks detected with TRβ-HAB (C-terminal tag) and HAx3-TRβ1 (N-terminal tag) described by Shabtai et al, 2021, main text, ref 29. In both conditions, ~70% of TRβ-HAB peaks overlap with HAx3-TRβ1 peaks (false discovery rate, FDR: 1e-6). The total numbers of peaks and background vary depending on different experimental approaches.

TRβ-HAB peaks (ChAP-seq) in hyper- and hypothyroid conditions identified by differential analysis of HAB and control BirA datasets in similar hyper- and hypothyroid conditions; GEO access # GSE133110.

HAx3-TRβ1 peaks (ChIP-seq) in hyper- and hypothyroid conditions identified by differential analysis with wild type controls using anti-HA ChIP-seq in euthyroid conditions; GEO access # GSE159648.

b, Examples of peaks near T3-induced and T3-suppressed liver genes (ref 29, main text)(IGV gene maps normalized using Wig files in SICER analysis. Normalized read scales, numbers on left of maps. Hypo- and hyperthyroid conditions were induced chemically using MMI (this study) for TRβ-HAB and propylthiouracil (PTU) for HAx3-TRβ1 (as described in ref. 29, main text).

a

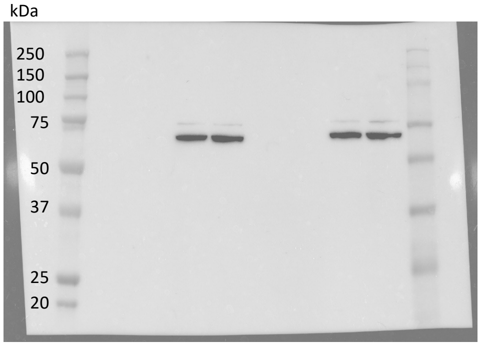

western blot, anti-lamin A/C

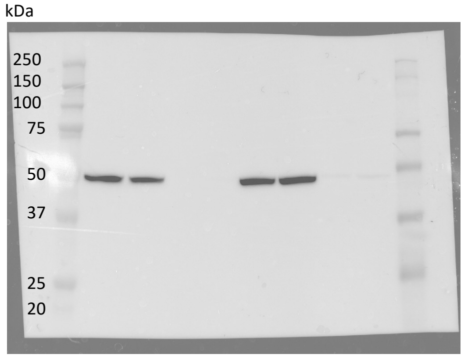

western blot, anti-tubulin-alpha

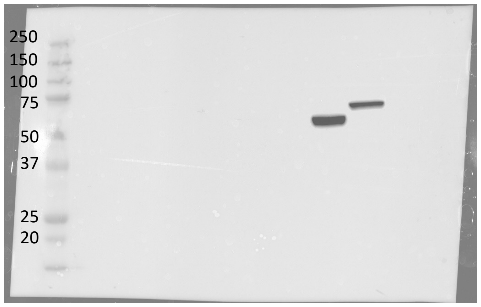

western blot, anti-TRβ2

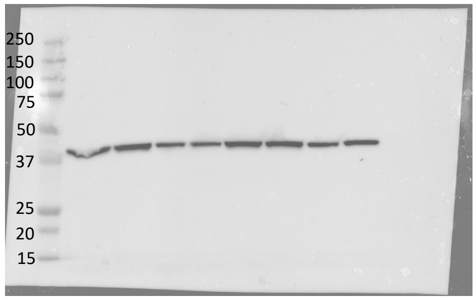

western blot, anti-actin-β

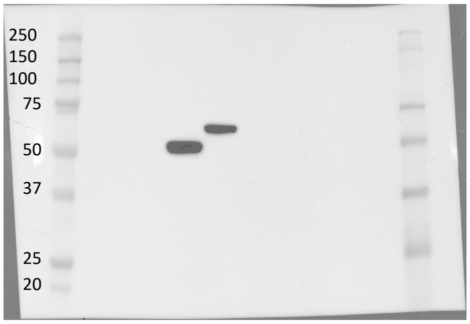

western blot, anti-TRβ1

b

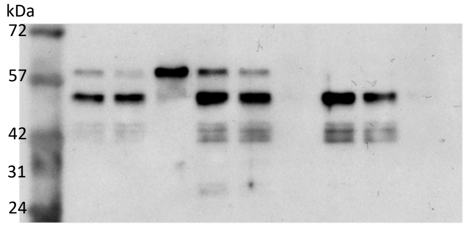

pull-down western blot: anti-TRβ2

Supplementary Fig 7. Scanned images of western blots with markers

a, Scanned images with markers for Supplementary Figure 1c; b, Scanned image with markers for Supplementary Figure 3a.

Supplementary Table 1. Gene ontology related to T3-regulated ATAC sites in pituitary chromatin (identified by comparison of hypothyroid versus hyperthyroid conditions)

**Gene ontology groups** (see Fig 3E)	**Genes**
regulation of generation of precursor metabolites and energy<br>P = 6.44E-10; count = 18/92	Adra1b, Akt2, Cavin3, Cox7a2l, Ddit4, Dyrk2, Fbp1, Grb10, Hdac4, Htr2a, Igf1, Irs2, Pomc, Ppara, Ppargc1a, Ppif, Slc25a33, Sorbs1
positive regulation of fatty acid metabolic process<br>P = 1.67E-08; count = 11/33	Akt2, Anxa1, Avpr1a, Elovl5, Ghsr, Irs2, Lpgat1, Nr4a3, Ppara, Ppargc1a, Ptgs2
cellular response to peptide hormone stimulus<br>P = 8.31E-08; count = 40/202	Actn2, Agtr1b, Akt2, Apc, Apobec1, Arg1, Arid1b, Baiap2l1, Cpeb2, Crhr2, Edn1, Eef2k, Foxo1, Fyn, Gclc, Gh, Ghr, Ghsr, Gjb2, Igf1, Igf1r, Inhbb, Irs2, Jak2,L pin1, Mup19, Mup5, Nfkb1, Nr4a3, Pdk4, Plcb1, Por, Pparg, Prkci, Rab31, Sgk1, Slc25a33, Sorbs1, Src, Trib3
cellular response to hormone stimulus<br>P = 1.45E-07; count = 92/452	Acod1, Actn2, Agtr1b, Akt2, Anxa1, Apc, Apob, Apobec1, Arg1, Arid1b, Atp1a1, Atp1a3, Atp2b1, Avpr1a, Baiap2l1, Bmp7, Carm1, Cga, Cpeb2, Crh, Crhr2, Ddit4, Edn1, Eef2k, Efna5, Egfr, Eif4e, Epha3, Esr1, Foxo1, Fyn, Gclc, Gclm, Gh, Ghr, Ghsr, Gja1, Gjb2, Gpr22, Hdac4, Igf1, Igf1r, Inhbb, Irs2, Jak2, Klf9, Lpin1, Med17, Mup19, Mup5, Ncoa1, Nfkb1, Npffr2, Nr2c1, Nr2f1, Nr3c1, Nr3c2, Nr4a3, Paqr8, Pdk4, Phex, Pid1, Plcb1, Por, Ppara, Pparg, Ppargc1a, Ppargc1b, Prkci, Prlr, Ptger4, Ptgfr, Rab31, Rarb, Rora, Rorb, Rxrg, Sfrp1, Sgk1, Slc25a33, Slit3, Sorbs1, Src, Sstr1, Sstr3, Stc1, Thrb, Trh, Trib3, Ucp3, Uri1, Zfp36l2
regulation of ATP metabolic process<br>P = 3.36E-07; count = 11/56	Cox7a2l, Ddit4, Fbp1, Hdac4, Htr2a, Igf1, Pid1, Ppara, Ppargc1a, Ppif, Slc25a33
cellular response to peptide<br>P = 1.29E-06; count = 47/242	Actn2, Agtr1b, Akt2, Apc, Apobec1, Arg1, Arid1b, Atp1a3, Baiap2l1, Cacna2d1, Cpeb2, Crhr2, Ddx11, Edn1, Eef2k, Epha4, Foxo1, Fyn, Gclc, Gh, Ghr, Ghsr, Gjb2, Igf1, Igf1r, Inhbb, Irs2, Jak2, Klf3, Klf5, Lpin1, Mup19 ,Mup5, Nfkb1, Nr4a3, Pdk4, Plcb1, Por, Pparg, Prkci, Rab31, Sgk1, Slc25a33, Sorbs1, Src, Tlr4, Trib3

Supplementary Table 2. Gene ontology related to TRβ-HAB binding sites in pituitary chromatin (untreated conditions)

**Gene ontology groups:** (see Fig 1E and Fig S3B)	**Genes**
cellular response to insulin stimulus<br>P = 4.31E-27; count = 75/131	Akt1 ,Akt2, Akt3, Apc, Apobec1, Appl1, Baiap2, Baiap2l1, Bcar1, C2cd5, Cav2, Ccl2, Cpeb1, Cpeb2, Cyfip1, Dennd4c, Eef2k, Errfi1, Fer, Foxc2, Foxo1, Gclc, Gh, Ghrhr, Ghsr, Got1, Grb2, Gsk3a, Gsk3b, Igf1r, Inhbb, Irs2, Irs3, Kl, Lpin1, Mup19, Mup5, Ndel1, Pck1, Pde3b, Pdk4, Pdpk1, Pik3r1, Pik3r2, Pkm, Plcb1, Ppat, Prkcz, Pten, Ptpn1, Rab31, Rab8a, Rhoq, Sgk1, Sh2b2, Sik2, Slc25a33, Slc2a4, Slc2a8, Slc9a1, Socs7, Soga1, Sorbs1, Src, Srebf1, Srsf3, Star, Stxbp4, Trib3, Tusc5, Ucp2, Wdr11, Wdtc1, Ywhag, Zfp36l1
cellular response to peptide hormone stimulus<br>P = 6.97E-26; count = 112/202	Actn2, Adcy6, Agtr1a, Agtr1b, Akt1, Akt2, Akt3, Apc, Apobec1, Appl1, Arg1, Arid1b, Baiap2, Baiap2l1, Bcar1, C2cd5, Car2, Cav2, Ccl2, Cpeb1, Cpeb2, Cps1, Creb1, Crhr2, Cyfip1, Dennd4c, Edn1, Eef2k, Errfi1, Fer, Foxc2, Foxo1, Fyn, Gcgr, Gclc, Gh, Ghrhr, Ghsr, Gjb2, Glp1r, Got1, Gper1, Gpr173, Grb2, Gsk3a, Gsk3b, Hsf1, Igf1, Igf1r, Inhbb, Irs2, Irs3, Itgb3, Jak2, Kl, Lpin1, Mdm2, Mup19, Mup5, Ndel1, Nfe2l2, Nfkb1, Nkx61, Nr4a1, Nr4a2, Nr4a3, Pck1, Pde3b, Pdk4 Pdpk1, Pik3r1, Pik3r2, Pkm, Plcb1, Por, Ppat, Prkcz, Pten, Ptk2, Ptpn1, Pxn, Rab31, Rab8a, Rangap1, Rap1b, Rhoq, Sgk1, Sh2b2, Sik2, Slc25a33, Slc2a4, Slc2a8, Slc9a1, Socs7, Soga1, Sorbs1, Src, Srebf1, Srsf3, Star, Stat5a, Stat5b, Stxbp4, Trib3, Tusc5, Ucp2, Umodl1, Wdr11, Wdtc1, Xbp1, Ywhag, Zfp36l1
regulation of generation of precursor metabolites and energy<br>P = 4.31E-27; count = 47/92	Adra1b, Akt1, Akt2, C1qtnf2, Cavin3, Cbfa2t3, Cisd1, Ddit4, Dyrk2, Fbp1, Gcgr, Gck, Grb10, Hdac4, Hif1a, Hmgb1, Htr2a, Ier3, Igf1, Irs2, Khk, Myog, Nkx11, Ogt, P2rx7, Park7, Pgam1, Phlda2, Pik3ca, Pnpt1, Pomc, Ppara, Ppargc1a, Ppif, Ppp1cb, Ppp1r3b,Prkaa2, Prkag2, Sirt6,Slc25a23, Slc25a33, Sorbs1, Tigar, Trap1, Trp53, Trpv4, Uqcc2
regulation of cellular ketone metabolic process by regulation of transcription from RNA polymerase II promoter<br>P = 4.31E-27; count = 2/2	Ncor2, Ppara
regulation of cellular carbohydrate metabolic process<br>P = 5.22E-16; count = 69/141	Acacb, Adcyap1r1, Adra1b, Akt1, Akt2, Arpp19, Bad, Bpgm, C1qtnf1, C1qtnf2, Cbfa2t3, Cd244, Ddit4, Dgat2, Dgkq, Dyrk2, Fbp1, Foxa2, Foxo1, Gcgr, Gck, Gper1, Grb10, Hdac4, Hmgb1, Ier3, Igf1, Igfbp4, Irs2, Khk, Lep, Lhcgr, Midn, Mlycd, Mup19, Mup5, Myog, Ncoa2, Nkx1-1, Nln, Nr1d1, Nr3c1, Ntsr1, Ogt, P2ry1, Park2, Pdk4, Pgam1, Phlda2, Pomc, Pou1f1, Ppara, Ppargc1a, Ppp1cb, Ppp1r3b, Prkce, Ranbp2, Rora, Sik1, Sirt6, Slc45a3, Soga1, Sorbs1, Src, Tcf7l2, Tff3, Tigar, Trp53, Ugt1a1

Supplementary Table 3, oligonucleotide primers.

Primer Name	sequence	Figure	Purpose
TRb-F	CCATGTGACACACTTTTGGC	Fig. 1. c; SI Fig. 1. b	RT-PCR; Thrb HAB Genotyping
TRb-WT-R	GTGCTGCAGGAATGACAAGA	Fig. 1. c; SI Fig. 1. b	RT-PCR; Thrb HAB Genotyping
Thrb-HAB-R	CATTACTCGTGCCACTCGATCTTC	Fig. 1. c; SI Fig. 1. b	RT-PCR; Thrb HAB Genotyping
Actb-F	ATGGAGGGGAATACAGCCC	Fig. 4. b	RT-PCR
Actb-R	TTCTTTGCAGCTCCTTCGTT	Fig. 4. b	RT-PCR
Myh6-F	GCCTGTGTACAATGCGGAAG	Fig. 4. b	RT-PCR
Myh6-R	CGGATTCTCCGGTGATGAGG	Fig. 4. b	RT-PCR
Bpifb3-F	CACGCTTGCTCGGATTGAC	Fig. 4. b	RT-PCR
Bpifb3-R	GCCACCGTAACTCAGCAGG	Fig. 4. b	RT-PCR
Tmem45b-F	ACCACAAGGGCTTGAAGAATAAC	Fig. 4. b	RT-PCR
Tmem45b-R	GGTGCAGGTGAGGTCCATC	Fig. 4. b	RT-PCR
Myh7-F	TGTCAACACTAAGAGGGTCATC	Fig. 4. b	RT-PCR
Myh7-R	TTGGATGATTTGATCTTCCAGGG	Fig. 4. b	RT-PCR
Ahrr-F	ACATACGCCGGTAGGAAGAGA	Fig. 4. b	RT-PCR
Ahrr-R	GGTCCAGCTCTGTATTGAGGC	Fig. 4. b	RT-PCR
C2cd4c-F	GGTTCTTGGAACGACTTCGGG	Fig. 4. b	RT-PCR
C2cd4c-R	CGGAGTCAGCACGTTGCTA	Fig. 4. b	RT-PCR
Lrat-F	CCGTCCCTATGAAATCAGCTC	Fig. 4. b	RT-PCR
Lrat-R	ATGGGCGACACGGTTTTCC	Fig. 4. b	RT-PCR
Bcl3-F	CCGGAGGCCCTTTACTACC	Fig. 4. b	RT-PCR
Bcl3-R	GGGTGAGTAGGCAGGTTCAG	Fig. 4. b	RT-PCR
Ceacam16-F	GACTCTTAACAGGCAGTTTCAGACGG	Fig. 4. b	RT-PCR
Ceacam16-R	AGACGAAGGGTATCCCTACGCTC	Fig. 4. b	RT-PCR
Ttbk1-F	GTGTGCAGGTTCATTGGCTG	Fig. 4. b	RT-PCR
Ttbk1-R	AACGTGAAAGTGCCCCTTGG	Fig. 4. b	RT-PCR
Dio3-F	TGTACCTGACCACCGTTCA	Fig. 4. b	RT-PCR
Dio3-R	GGTGCACCTTGTTGTAGTACTC	Fig. 4. b	RT-PCR
Gh-F	GCTACAGACTCTCGGACCTC	Fig. 4. b	RT-PCR
Gh-R	CGGAGCACAGCATTAGAAAACAG	Fig. 4. b	RT-PCR
Prl-F	CAGGGGTCAGCCCAGAAAG	Fig. 4. b	RT-PCR
Prl-R	TCACCAGCGGAACAGATTGG	Fig. 4. b	RT-PCR
Trhde-F	AGTCCGATTATATGCGAGACCC	Fig. 4. b	RT-PCR
Trhde-R	GGATGCTTAGGCACAGCTAAAAG	Fig. 4. b	RT-PCR
Foxo6-F	GTGGGGGAACCTTTCCTACG	Fig. 4. b	RT-PCR
Foxo6-R	TTCTGCACGCGGATGAACC	Fig. 4. b	RT-PCR
Hr-F	CCCCTGTGAACGGCATTGT	Fig. 4. b	RT-PCR
Hr-R	CCCCTCCAAAAGGGAGCAG	Fig. 4. b	RT-PCR
Tyrp1-F	CCCCTAGCCTATATCTCCCTTTT	Fig. 4. b	RT-PCR
Tyrp1-R	TACCATCGTGGGGATAATGGC	Fig. 4. b	RT-PCR
Anxa10-F	ATGTTTTGCGGGGAATATGTCC	Fig. 4. b	RT-PCR
Anxa10-R	CAATCAGCATGTCTTTGTTGCAG	Fig. 4. b	RT-PCR
Gc-F	CCTGCTGGCCTTAGCCTTT	Fig. 4. b	RT-PCR
Gc-R	TGCTCAAATGTGCTACTGGAAA	Fig. 4. b	RT-PCR
Ptpn5-F	ATGTGCTGTAGTGAGAGGCTG	Fig. 4. b	RT-PCR
Ptpn5-R	TGGTGGCATCTCTTTCTGCTT	Fig. 4. b	RT-PCR
Slc22a19-F	AAATGCAGATCCTGCGTGTATT	Fig. 4. b	RT-PCR
Slc22a19-R	CCTAAAGCAGTTGCCCTGATTA	Fig. 4. b	RT-PCR
Th-F	GTCTCAGAGCAGGATACCAAGC	Fig. 4. b	RT-PCR
Th-R	CTCTCCTCGAATACCACAGCC	Fig. 4. b	RT-PCR
Trhr-F	GCATCCATAAAAGGGCAAAC	Fig. 4. b	RT-PCR
Trhr-R	TAAATGCCACCAACAGATGC	Fig. 4. b	RT-PCR
Dio2-F	ACACTGGAATTGGGAGCATC	Fig. 4. b	RT-PCR
Dio2-R	ATGCTGACCTCAGAAGGGCT	Fig. 4. b	RT-PCR
Cga-F	AAGCTAGGAGCCCCCATCTAC	Fig. 4. b	RT-PCR
Cga-R	ACTCTGGCATTTCCCATTACTG	Fig. 4. b	RT-PCR
Tshb-F	GGGCAAGCAGCATCCTTTTG	Fig. 4. b	RT-PCR
Tshb-R	GTGTCATACAATACCCAGCACAG	Fig. 4. b	RT-PCR
Southern blot probe 3'-end-F	TGCCGCCTTCAAGAGCAATA	SI Fig. 1. a	Making Southern blot probe
Southern blot probe 3'-end-R	TTTCTGGTAGGCAAGCCCTG	SI Fig. 1. a	Making Southern blot probe
Thrb1-prom-A-Nhe-F	TACGCAGCTAGCAGCTGCTGGGAGAATCGAAC	SI Fig. 4. c	NheI, Luciferase assay
Thrb1-Prom-A-H3-R	TACGCAaagctTACTGGCATTCCCTCTGACC	SI Fig. 4. c	HindIII, Luciferase assay
Thrb1-enhancer-BH-402F	TACGCAGGATCCATGCCACTTCTGTGAGTGTTC	SI Fig. 4. c	BamHI, Luciferase assay
Thrb1-enhancer-Sal-793R	TACGCAGTCGACAGGCAAGATTTTCTGCAGGC	SI Fig. 4. c	SalI, Luciferase assay
Thrb1-sitemutated-F	GAACACCCCAGTGTAATCAGATGAAACAGCATATACACTTACAAGGCCTGC	SI Fig. 4. b c	Luciferase assay
Thrb1-sitemutated-R	GTGTATATGCTGTTTCATCTGATTACACTGGGGTGTTCATTTTAAAATTAG	SI Fig. 4. b c	Luciferase assay
Prom-B-Xho-F	TACGCACTCGAGCAGCGCTCTCTGTCCTCTTG	SI Fig. 4. c	XhoI, Luciferase assay
Prom-B-H3-R	TACGCAaagcttGCCGCAAAGGGTGAACTG	SI Fig. 4. c	HindIII, Luciferase assay
Ceacam16-enhancer-BglI-F	TACGCAAGATCTCGGTGTGTCAATCGGAGAGT	SI Fig. 4. c	BglII, Luciferase assay
Ceacam16-enhancer-XhoI-R	TACGCACTCGAGGTAGAGCCCTTGTGCTGAGG	SI Fig. 4. c	XhoI, Luciferase assay
Ceacam16-sitemutated1-F	GAAGATACTAATTTCGATTTGTTACAAGGTGCCTGAGTCAGAGG	SI Fig. 4. b c	Luciferase assay
Ceacam16-sitemutated1-R	CTCCTTCAACTGTGAAATTACCTCAAATTGCTCTCCCTGTCTG	SI Fig. 4. b c	Luciferase assay
Ceacam16-sitemutated2-F	GAAGATACTAATTTCGATTTGTTACAAGGTGCCTGAGTCAGAGG	SI Fig. 4. b c	Luciferase assay
Ceacam16-sitemutated2-R	CAGGCACCTTGTAACAAATCGAAATTAGTATCTTCCCAGACCTG	SI Fig. 4. b c	Luciferase assay
Ceacam16-sitemutated3-F	TCCCCTTGATTGCAGCCCATTACACAGTGATACTCTTGAGGAAC	SI Fig. 4. b c	Luciferase assay
Ceacam16-sitemutated3-R	AGTATCACTGTGTAATGGGCTGCAATCAAGGGGAAGGAGGGCTG	SI Fig. 4. b c	Luciferase assay
Thrb1-EcoRI-F	GATCgaattcTGGCCACCATGACTCCTAACAGTATGACAG		cloning pcDNA3-TRb1 and pcDNA3-TRb1-HAB
Thrb-HAB-XhoI-R	GATCCTCGAGtcattactcgtgccactcgatc		cloning pcDNA3-TRb1-HAB and pcDNA3-TRb2-HAB
Thrb-XhoI-R	GATCCTCGAGctcgagTTAGTCCTCAAATACTTCTAAG		cloning pcDNA3-TRb1 and pcDNA3-TRb2
Thrb2-EcoRI-F	GATCGAATTCTGGCCACCatgaactactgtatgccagagGTAC		cloning pcDNA3-TRb2 and pcDNA3-TRb2-HAB
BirA-F	TTCAGACACTGCGTGACT		Rosa26 BirA Genotyping
BirA-R	AGTCAGCTCCACCAGAATGC		Rosa26 BirA Genotyping
WT-F	GTGTAACTGTGGACAGAGGAG		Rosa26 BirA Genotyping
WT-R	ACCACTGGCTGGCTAAACTC		Rosa26 BirA Genotyping
Sun1-com-F	GCACTTGCTCTCCCAAAGTC		CAG-Sun1/sfGFP Genotyping
Sun1-R	GTTATGTAACGCGGAACTCC		CAG-Sun1/sfGFP Genotyping
Sun1-wt-R	CATAGTCTAACTCGCGACACTG		CAG-Sun1/sfGFP Genotyping
Thrb-KO-P650-M4F	GCACAGGCAGGAAGTAGGCTGTTC		Thrb KO Genotyping
Thrb-KO-M4R	GCTGTGGAGCAAACTCGTCTCCTC		Thrb KO Genotyping
Thrb-KO-NEO2300R	GCCAGCGGGGCTGCTAAAG		Thrb KO Genotyping

Supplementary Table 4, antibodies and mouse strains.

Product Name	Manufacturer	Cat #	RRID	
Anti-TRβ2 antibody produced in rabbit	DF lab		AB_297439	Fig. 1 (immunostain)
				Sup. Fig. 1 (Western blot)
Anti-acetyl-alpha tubulin Antibody, clone 6-11B-1	Millipore Sigma	MABT868	AB_2819178	Fig. 1 D, (immunostain)
IgG Rabbit Polyclonal Antibody	Sigma-Aldrich	PP64	AB_97852	Fig. 2 (ChIP)
Anti-trimethyl-Histone H3 (Lys4) Antibody (H3K4me3)	Sigma-Aldrich	07-473	AB_1977252	Fig. 2 (ChIP)
Anti-Histone H3 (mono methyl K4) antibody (H3K4me1)	Abcam	ab8895	AB_306847	Fig. 2 (ChIP)
Anti-Histone H3 (acetyl K27) antibody (H3K27ac)	Abcam	ab4729	AB_2118291	Fig. 2 (ChIP)
Anti-GFP antibody	Abcam	ab290	AB_303395	Fig. 6 (ATAC)
Anti-TRβ1 antibody produced in rabbit	DF lab			Sup. Fig. 1 (Western blot)
Anti-Actin Antibody, clone C4	Millipore Sigma	MAB1501	AB_2223041	Sup. Fig. 1 (Western blot)
Anti-alpha-Tubulin (DM1A) Mouse mAb	Cell Signaling Technology	3873	AB_1904178	Sup. Fig. 1 (Western blot)
Anti-Lamin A/C (4C11) mouse monoclonal antibody	Cell Signaling Technology	4777	AB_10545756	Sup. Fig. 1 (Western blot)
Anti-rabbit IgG (Conformation Specific) (L27A9) mAb (HRP Conjugate)	Cell Signaling Technology	5127	AB_10892860	Sup. Fig. 1 (Western blot)
Anti-Mouse IgG (Light Chain Specific) (D3V2A) mAb (HRP Conjugate)	Cell Signaling Technology	58802	AB_2799549	Sup. Fig. 1 (Western blot)
Goat anti-Rabbit IgG (H+L)-HRP Secondary Antibody	Thermo Fisher Scientific	G-21234	AB_2536530	Sup. Fig. 1 F (Western blot)
Goat Anti-Chicken IgG (H+L)-Alexa Fluor-488 Secondary Antibody	Thermo Fisher Scientific	A-11039	AB_2534096	Sup. Fig. 2 B (immunostain)
Anti-Cga Polyclonal Antibody produced in rabbit	Thermo Fisher Scientific	PA5-119024	AB_2903524	Sup. Fig. 2 C (immunostain)
Anti-Tshb Polyclonal Antibody produced in rabbit	Millipore Sigma	AB976	AB_2208242	Sup. Fig. 2 C (immunostain)
Anti-Gh Polyclonal Antibody produced in rabbit	Millipore Sigma	AB940	AB_2111262	Sup. Fig. 2 C (immunostain)
Anti-Prl Polyclonal Antibody produced in rabbit	Millipore Sigma	AB960	AB_11210349	Sup. Fig. 2 C (immunostain)
Anti-Acth Polyclonal Antibody produced in rabbit	Millipore Sigma	AB902	AB_2166019	Sup. Fig. 2 C (immunostain)
Goat anti-Rabbit IgG (H+L)-Alexa Fluor 568 Secondary Antibody	Thermo Fisher Scientific	A-11011	AB_143157	Sup. Fig. 2 C (immunostain)
Anti-beta Galactosidase polyclonal antibody produced in chicken	Abcam	ab9361	AB_307210	Sup. Fig. 2 B (immunostain)
Thrb HAB mice	DF lab	none	none	
Thrb2-Cre mice	DF lab	none	none	
Wildtype C57BL/6J mice	Jackson Laboratory	000664	IMSR_JAX:000664	
Rosa26 Sun1-GFP mice	Jackson Laboratory	030952	IMSR_JAX:030952	
Rosa26-BirA mice	Jackson Laboratory	010920	IMSR_JAX:010920	
Thrb knock-out mice	DF lab / Jackson Laboratory	003462	IMSR_JAX:003462	

Supplementary Table 5.
Bam files of sequence datasets
and numbers of mapped reads.

File Name	Size	# of mapped reads	
**ChAPseq**			
Brain_untreated_ChAP_BirA123_control.bam	917.7 MB	17621949	Fig1. E. F.
Brain_untreated_ChAP_BirA1.bam	308.3 MB	2776568	rep 1
Brain_untreated_ChAP_BirA2.bam	290.2 MB	5717024	rep 2
Brain_untreated_ChAP_BirA3.bam	334.5 MB	5279482	rep 3
Brain_untreated_ChAP_HAB123_merged.bam	902.1 MB	16552540	Fig1. E. F.
Brain_untreated_ChAP_HAB1.bam	413.3 MB	6625443	rep 1
Brain_untreated_ChAP_HAB2.bam	338 MB	7714825	rep 2
Brain_untreated_ChAP_HAB3.bam	167.7 MB	6061147	rep 3
Pit_untreated_ChAP_BirA123_control.bam	3.85 GB	83593207	Fig1. E. F.
Pit_untreated_ChAP_BirA1.bam	2.06 GB	43958017	rep 1
Pit_untreated_ChAP_BirA2.bam	871.6 MB	19475904	rep 2
Pit_untreated_ChAP_BirA3.bam	910.2 MB	20159286	rep 3
Pit_untreated_ChAP_HAB123_merged.bam	2.82 GB	65016698	Fig1. E. F.
Pit_untreated_ChAP_HAB1.bam	1.03 GB	25632181	rep 1
Pit_untreated_ChAP_HAB2.bam	917.6 MB	20337322	rep 2
Pit_untreated_ChAP_HAB3.bam	865.7 MB	19047195	rep 3
**ChAPseq**			
Pit_mmi_ChAP_HAB_merged.bam	3.2 GB	67291047	mmi-TRb-HAB
Pit_mmi_ChAP_TRb_HAB1.bam	1.95 GB	40517813	rep 1
Pit_mmi_ChAP_TRb_HAB3.bam	1.21 GB	26773234	rep 2
Pit_mmi_ChAP_BirA1.bam	1.88 GB	45625638	rep 1
Pit_mmi_ChAP_BirA2.bam	1.9 GB	45240372	rep 2
Pit_T3_ChAP_HAB_merged.bam	4.07 GB	88903906	T3-TRb-HAB
Pit_T3_ChAP_TRb_HAB1.bam	1.92 GB	40974735	rep 1
Pit_T3_ChAP_TRb_HAB3.bam	2.1 GB	47929171	rep 2
Pit_T3_ChAP_BirA1.bam	1.98 GB	48975553	rep 1
Pit_T3_ChAP_BirA2.bam	1.37 GB	30651179	rep 2
**ChIPseq**			
Pit_mmi_ChIP_H3K27ac_merged.bam	1.44 GB	26884594	mmi-H3K27ac
Pit_mmi_ChIP_H3K27ac_1.bam	584.8 MB	10247593	rep 1
Pit_mmi_ChIP_H3K27ac_2.bam	357 MB	6347927	rep 2
Pit_mmi_ChIP_H3K27ac_3.bam	525.5 MB	10289074	rep 3
Pit_mmi_ChIP_H3K4m1_merged.bam	1.4 GB	24878286	mmi-H3K4me1
Pit_mmi_ChIP_H3K4m1_1.bam	517.6 MB	8589854	rep 1
Pit_mmi_ChIP_H3K4m1_2.bam	436.4 MB	7840774	rep 2
Pit_mmi_ChIP_H3K4m1_3.bam	461.5 MB	8447658	rep 3
Pit_mmi_ChIP_H3K4m3_merged.bam	1.86 GB	36922218	mmi-H3K4me3
Pit_mmi_ChIP_H3K4m3_1.bam	652.2 MB	12338092	rep 1
Pit_mmi_ChIP_H3K4m3_2.bam	744 MB	14455368	rep 2
Pit_mmi_ChIP_H3K4m3_3.bam	496.4 MB	10128758	rep 3
Pit_mmi_ChIP_IgG_1.bam	426.4 MB	7953199	rep 1
Pit_mmi_ChIP_IgG_2.bam	605.5 MB	12357592	rep 2
Pit_mmi_ChIP_IgG_3.bam	293.8 MB	5992333	rep 3
Pit_T3_ChIP_H3K27ac_merged.bam	2.22 GB	45603040	T3-H3K27ac
Pit_T3_ChIP_H3K27ac_1.bam	899.8 MB	18895059	rep 1
Pit_T3_ChIP_H3K27ac_2.bam	1.22 GB	24719868	rep 2
Pit_T3_ChIP_H3K27ac_3.bam	112.3 MB	1988113	rep 3
Pit_T3_ChIP_H3K4m1_merged.bam	2.54 GB	50322876	T3-H3K4me1
Pit_T3_ChIP_H3K4m1_1.bam	426.9 MB	7652335	rep 1
Pit_T3_ChIP_H3K4m1_2.bam	973 MB	19838894	rep 2
Pit_T3_ChIP_H3K4m1_3.bam	1.18 GB	22831647	rep 3
Pit_T3_ChIP_H3K4m3_merged.bam	2.03 GB	42305478	T3-H3K4me3
Pit_T3_ChIP_H3K4m3_1.bam	592.4 MB	11595791	rep 1
Pit_T3_ChIP_H3K4m3_2.bam	1.14 GB	25020923	rep 2
Pit_T3_ChIP_H3K4m3_3.bam	304.1 MB	5688764	rep 3
Pit_T3_ChIP_IgG_1.bam	679.9 MB	15777400	rep 1
Pit_T3_ChIP_IgG_2.bam	540.2 MB	11977435	rep 2
Pit_T3_ChIP_IgG_3.bam	1.06 GB	23911875	rep 3
**ATACseq**			
Pit_mmi_Sun_ATAC_merged.bam	6.13 GB	116405516	mmi_Sun_ATAC
Pit_mmi_Sun_ATAC_1.bam	1.48 GB	30727842	rep 1
Pit_mmi_Sun_ATAC_2.bam	1.15 GB	20189846	rep 2
Pit_mmi_Sun_ATAC_3.bam	2.01 GB	37056251	rep 3
Pit_mmi_Sun_ATAC_4.bam	1.51 GB	28431577	rep 4
Pit_T3_Sun_ATAC_merged.bam	6.05 GB	120449023	T3_Sun_ATAC
Pit_T3_Sun_ATAC_1.bam	1.28 GB	27585498	rep 1
Pit_T3_Sun_ATAC_2.bam	1.31 GB	29355326	rep 2
Pit_T3_Sun_ATAC_3.bam	1.43 GB	24870694	rep 3
Pit_T3_Sun_ATAC_4.bam	2.06 GB	38637505	rep 4
**RNAseq**			
RNA_Pit_WT_mmi_merged.bam	4.45 GB	97167290	mmi-RNAseq-coverage
RNA-mmi-WT1.bam	2.28 GB	49870290	rep 1
RNA-mmi-WT2.bam	2.36 GB	51218535	rep 2
RNA-mmi-WT3.bam	1.87 GB	40419320	rep 3
RNA-mmi-WT4.bam	1.26 GB	26617924	rep 4
RNA_Pit_WT_T3_merged.bam	4.21 GB	93115032	T3-RNAseq-coverage
RNA-T3-WT1.bam	2.97 GB	66298984	rep 1
RNA-T3-WT2.bam	2.54 GB	55575068	rep 2
RNA-T3-WT3.bam	1.11 GB	24012377	rep 3
RNA-T3-WT4.bam	1.71 GB	36576093	rep 4
RNA-mmi-ThrbKO1.bam	1.95 GB	42544505	rep 1
RNA-mmi-ThrbKO2.bam	2.12 GB	45672371	rep 2
RNA-mmi-ThrbKO3.bam	2.6 GB	56244283	rep 3
RNA-mmi-ThrbKO4.bam	1.5 GB	30362477	rep 4
RNA-mmi-ThrbKO5.bam	1.55 GB	31213200	rep 5
RNA-T3-ThrbKO1.bam	2.5 GB	55267627	rep 1
RNA-T3-ThrbKO2.bam	2.55 GB	55765969	rep 2
RNA-T3-ThrbKO3.bam	2 GB	40866658	rep 3
RNA-T3-ThrbKO4.bam	1.58 GB	32021562	rep 4
RNA-T3-ThrbKO5.bam	1.06 GB	20885954	rep 5
