# Supplementary material for: Thyroid hormone-regulated chromatin landscape and transcriptional sensitivity of the pituitary gland
Source: Commun Biol. 2023 Dec 11;6:1253. doi: 10.1038/s42003-023-05546-y (PMC10713718; doi:10.1038/s42003-023-05546-y)
Supplement: Supplementary file 2 — Description of Additional Supplementary Files [file 42003_2023_5546_MOESM2_ESM.pdf]

Description of Additional Supplementary Files

File name: Supplementary Data
Description: Source data for graphs included in the manuscript.
